# Changing Ecological Opportunities Facilitated the Explosive Diversification of New Caledonian *Oxera* (Lamiaceae)

**DOI:** 10.1093/sysbio/syy070

**Published:** 2018-10-26

**Authors:** Laure Barrabé, Sébastien Lavergne, Giliane Karnadi-Abdelkader, Bryan T Drew, Philippe Birnbaum, Gildas Gâteblé

**Affiliations:** 1 Institut Agronomique néo-Calédonien (IAC), Equipes ARBOREAL and SOLVEG, BP 711, Mont-Dore 98810, New Caledonia; 2 Endemia, Plant Red List Authority, 7 rue Pierre Artigue, Nouméa 98800, New Caledonia; 3 Laboratoire d’Ecologie Alpine, CNRS - Université Grenoble Alpes, UMR 5553, Grenoble F-38000, France; 4 Department of Biology, University of Nebraska-Kearney, Kearney, NE 68849, USA; 5 UMR AMAP, Université de Montpellier, CIRAD, CNRS, INRA, IRD, Montpellier 34398, France

**Keywords:** Adaptive radiation, allopatry, leapfrog radiation, Lamiaceae, New Caledonia, niche shifts, Oxera

## Abstract

Phylogenies recurrently demonstrate that oceanic island systems have been home to rapid clade diversification and adaptive radiations. The existence of adaptive radiations posits a central role of natural selection causing ecological divergence and speciation, and some plant radiations have been highlighted as paradigmatic examples of such radiations. However, neutral processes may also drive speciation during clade radiations, with ecological divergence occurring following speciation. Here, we document an exceptionally rapid and unique radiation of Lamiaceae within the New Caledonian biodiversity hotspot. Specifically, we investigated various biological, ecological, and geographical drivers of species diversification within the genus *Oxera*. We found that *Oxera* underwent an initial process of rapid cladogenesis likely triggered by a dramatic period of aridity during the early Pliocene. This early diversification of *Oxera* was associated with an important phase of ecological diversification triggered by significant shifts of pollination syndromes, dispersal modes, and life forms. Finally, recent diversification of *Oxera* appears to have been further driven by the interplay of allopatry and habitat shifts likely related to climatic oscillations. This suggests that *Oxera* could be regarded as an adaptive radiation at an early evolutionary stage that has been obscured by more recent joint habitat diversification and neutral geographical processes. Diversification within *Oxera* has perhaps been triggered by varied ecological and biological drivers acting in a leapfrog pattern, but geographic processes may have been an equally important driver. We suspect that strictly adaptive radiations may be rare in plants and that most events of rapid clade diversification may have involved a mixture of geographical and ecological divergence.

Oceanic islands are widely regarded as laboratories of evolution mainly owing to their isolated past biogeographical history (Losos and Ricklefs 2009). Their geographical isolation limits dispersal events from outside, resulting in ecological niches being filled by species diversification rather than colonization. Consequently, islands are expected to have sheltered many adaptive radiations, where single lineages have diversified rapidly into several distinct niches, resulting in disproportionately high phenotypic diversity (Schluter 2000) when compared with their continental relatives (Silvertown et al. 2005). This phenomenon can be particularly apparent on large and high-elevation islands that exhibit diverse and sharp environmental gradients (Whittaker et al. 2008). Although adaptive radiations may not necessarily result from an acceleration of speciation rates (see Givnish 2015 for a discussion), all adaptive radiations are spurred by ecological opportunities; that is, the release of vacant niches due to unused resources, species extinctions or the acquisition of new traits (Losos 2010). The existence of adaptive radiations thus posits a central role of natural selection causing ecological divergence and speciation, and some plant radiations have been highlighted repeatedly as paradigmatic examples of this scenario, such as the silversword alliance and lobeliads in Hawaii (Carlquist et al. 2003; Givnish et al. 2009), the Macaronesian *Aeonium* and *Echium* (Jorgensen and Olesen 2001), and *Veronica* (*Hebe*) in New Zealand (Wagstaff and Garnock-Jones 1998).

However, adaptive radiations *sensu stricto* appear to be quite uncommon in the plant kingdom, with the aforementioned well-documented cases probably representing rare exceptions. In fact, many radiations likely occur, at least in part, in a non-adaptive way (Givnish 1997, Rundell and Price 2009). Under such a scenario, speciation is not primarily driven by ecological divergence, but mainly by neutral divergence in allopatry. Ecological differentiation may occur following non-ecological speciation, through random trait divergence, adaptation to different climatic regimes or habitats, or due to trait divergence favoring local coexistence if secondary sympatry occurs (e.g., Tobias et al. 2014). While molecular phylogenies will often provide little power to discriminate between different scenarios, it remains possible to assess the relative importance of ecological differentiation and geographic divergence in rapid species diversifications. Considering that theoretical evidence suggests that most rapid evolutionary radiations may have occurred through a mixture of ecological speciation and neutral divergence in allopatry (Aguilée et al. 2012, 2011), we can legitimately wonder whether purely adaptive radiations have ever occurred in plants.

The New Caledonian biodiversity hotspot is a remote archipelago in the southwest Pacific, composed of an old-, large-, and high-elevation main island (= Grande Terre, ca. 37 Ma, ca. 16,600 km$^{2}$, 1600 m max. elevation) exhibiting exceptional levels of species richness and endemism (ca. 3400 species, 75% endemic; Morat et al. 2012; Munzinger et al. 2016). The singular geological and climatic New Caledonian history led to the implementation of complex and abrupt environmental gradients, resulting in a mosaic of highly distinct habitats. The archipelago topography is especially complex on Grande Terre, with many relatively steep valleys and high summits (Bonvallot 2012) presenting both physical barriers to dispersal and strong selection gradients. The archipelago also has a suite of diverse bedrock types, in particular volcano-sedimentary, metamorphic and ultramafic (metal-rich soils with chemical and physical properties that constrain plant growth). These latter bedrock types, which act as strong environmental filters and exhibit a patchy distribution, have probably played a key role in plant speciation and flora evolution (Pillon et al. 2010). In addition, Pliocene and Pleistocene climatic fluctuations considerably affected the dynamic of New Caledonian biotas, leading to the origination of new habitats such as the unique shrubby sclerophyllous vegetation (i.e., maquis; Jaffré 1980), during periods of forest contraction (Hope and Pask 1998; Stevenson 2004; Stevenson and Hope 2005). This likely triggered speciation in palms (Pintaud et al. 2001), and has contributed to the persistence of old angiosperm lineages in forest refugia (Pouteau et al. 2015). All the aforementioned characteristics have contributed to the tremendous plant diversity of New Caledonia (Jaffré 1993), and have also driven numerous plant radiations (e.g., *Psychotria*; *Pycnandra*; Munzinger et al. 2016). Though plant radiations are increasingly highlighted in the archipelago through molecular investigations, few studies have focused on the relative effect of drivers implied in their *in situ* diversification (e.g., Barrabé et al. 2014; Pillon et al. 2014; Paun et al. 2016).

The old age, geographic isolation, and geologic and topographic complexity of New Caledonia suggest that adaptive radiations may be common among many genera endemic to New Caledonia. However, few of these genera exhibit the joint signatures of morphological and ecological divergence expected from adaptive radiations, and rather seem to be relictual lineages (Pillon et al. 2017). The woody genus *Diospyros* (Ebenaceae) was the first purported clear case of an adaptive radiation of a plant group within New Caledonia due to its wide ecological diversity (Paun et al. 2016), although the radiation was not related to obvious morphological and/or physiological differences. The tree genus *Geissois* (Cunoniaceae) was described as a cryptic adaptive radiation, in which species exhibit notable differences in leaf element composition likely linked to the occupation of varied soils (Pillon et al. 2014), but does not fully satisfy the criteria of an adaptive radiation as outlined by Givnish (2015), as no physiological adaptation has yet been highlighted. It thus seems that New Caledonia has harbored few, if any, adaptive radiations in a strict sense (sensu Givnish 2015 and Schluter 2000). This has been explained as a consequence of the relatively old age of the island, the prevalence of woody species, the paucity of potential pollinator species, and/or a reduced number of ecological opportunities as compared with other island systems (Pillon et al. 2017).

In this work, we report on a molecular systematic investigation of the genus *Oxera* (Lamiaceae), which has been hypothesized to have diversified in New Caledonia through adaptive radiation (Pillon et al. 2014). The genus is composed of 33 species endemic to New Caledonia (Gâteblé unpublished data, available at http://endemia.nc/flore/fiche588), and was recently enlarged to include five other Australasian and Pacific species (Barrabé et al. 2015). It was demonstrated that the entire New Caledonian clade originated from a single colonization and constitutes the tenth largest plant radiation in the archipelago, which is puzzling given that Lamiaceae are an under-represented family within the flora of New Caledonia (Pillon et al. 2010). Distinct subclades were partially circumscribed within *Oxera* and can arguably be considered as several independent micro-radiations within the genus (Barrabé et al. 2015). *Oxera* exhibits strongly divergent morphology in terms of life form, flowers and fruits, and occupies a vast diversity of distinct habitats. In relation to this remarkable morphological diversity, we hypothesize that shifts of functional, reproductive (pollination and dispersal), and environmental niches may have played a key role during the diversification of *Oxera*. This role can be two-fold: 1) niche shifts may have triggered species differentiation and speciation, and 2) certain niche types may have accelerated the rate of genetic divergence and speciation.

The dazzling morphological and ecological diversity of New Caledonian *Oxera* could reflect adaptations to different ecological factors (see Givnish 2010 for a discussion of life form adaptations). First, the great array of life forms observed in *Oxera* could be associated with different environment preferences, as observed in the silversword alliance (Carpenter et al. 2003), and this may have created conditions that facilitated parapatric isolation and speciation. Robust lianas mainly grow in closed rainforests with leaves usually reaching the canopy, while slender lianas are mostly encountered in open sclerophyllous vegetation and forest edges; monocaulous trees (i.e., single-stemmed) are mainly confined to rainforest understories; and finally, shrub species are often able to establish in both closed and more open vegetation (Gâteblé, pers. obs.; see Fig. 1a and b). Such contrasting life forms clearly reflect functional strategies regarding growth under different light conditions (Givnish 1995; Santiago and Wright 2007; Selaya and Anten 2008).

**Figure 1.**
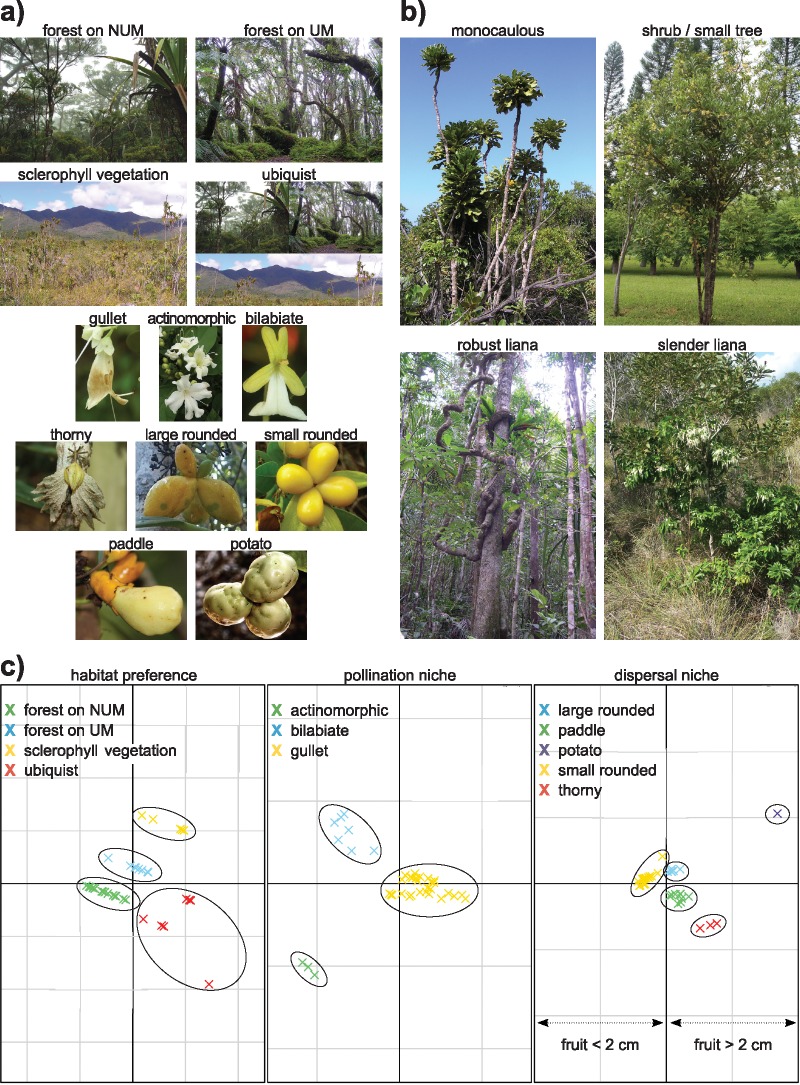
Biological and ecological data sets of *Oxera*. a) Illustration of habitat preferences and biological syndromes, from top to bottom: habitat preferences (UM = ultramafic soils; NUM $=$ non-ultramafic soils), pollination niche, and dispersal niche. b) Illustration of life forms. c) Niche syndromes inferred from the Hill and Smith principal component method, according to the first two axes of multivariate analyses.

Second, most species of *Oxera* are confined to narrow environmental conditions within New Caledonia. They occur either in rainforests, dry forests or maquis, or occur at different elevations and/or on different bedrock types (limestone, ultramafic, and volcano-sedimentary rocks). Although some species of *Oxera* are clearly specialized toward particular habitats or have very limited geographic ranges in narrow valleys or on remote summits, others are encountered throughout the archipelago due to wider environmental tolerances. These contrasting habitat and geographic patterns may be hypothesized as the signature of parapatric or allopatric speciation processes, and provide an interesting basis for testing whether habitat or geography (or both) have been major drivers of clade diversification.

Third, the floral diversity of *Oxera* was previously linked to distinct flower pollinators (de Kok 1998) based upon field observations of animal visits (Table 1).

**Table 1.** *In situ* direct observations of flower visitors and fruit consumers of *Oxera*

		Flowers		Fruits			
Subclade	Species	Flower visitor	Flower type (multivariate analyses)	Fruit consumer	Fruit type (multivariate analyses)	Maximum mericarp length	References
Australasian *Oxera*	*Oxera splendida*		Actinomorphic	Cassowaries, flying foxes, rats	Potato	62.5	Richards (1990), Hutson et al. (1992), Cooper and Cooper (2004)
Pacific *Oxera*	*Oxera amicorum*	Honey eater (*Myzomela cardinalis*, *Gymnomyza viridis*)	Actinomorphic	Columbidae (*Ducula pacifica*, *Didunculus strigirostris*), flying foxes	Rounded	41.7	Belcher and Sibson (1982), de Kok (1997), Beichle (1987), Trail (1994), Pillon (pers. obs.)
Pacific *Oxera*	*Oxera lehuntei*	Honey eater	Actinomorphic		Rounded	30.5	de Kok (1997)
“*baladica*” subclade	*Oxera aff. nuda*				Paddle	40.6	
“*baladica*” subclade	*Oxera baladica*	Honey eater (*Glycifohia undulata*), Zosteropidae (*Zosterops* sp.)	Gullet		Paddle	26.3	de Kok (1997), Fleurot (pers. obs.)
“*baladica*” subclade	*Oxera doubetiae* sp.nov.ined.				Thorny	40.6	
“*baladica*” subclade	*Oxera garoensis* sp.nov.ined.						
“*baladica*” subclade	*Oxera ounemoae* sp.nov.ined.				Thorny	30.6	
“*baladica*” subclade	*Oxera papineaui* sp.nov.ined.				Paddle	25.9	
“*baladica*” subclade	*Oxera sessilifolia*				Thorny	40.4	
“*baladica*” subclade	*Oxera vanuatuensis*	Birds	Gullet		Paddle	35.0	de Kok (1997)
“*neriifolia*” subclade	*Oxera neriifolia*	Pieridae (*Anaphaeis java*, *Eurema hecabe*), Sphingidae (*Gnathothlibus erotus*, *Hippotion scrofa*, *Hippotion celerio*, *Psilogramma lifuensis*, *Compsulyx cochereau*i, etc.)	Bilabiate		Rounded	14.8	de Kok (1997), Gâteblé and Ouriet (pers. obs.)
“*neriifolia*” subclade	*Oxera ovata*	Pieridae (*Anaphaeis java*), Sphingidae (*Gnathothlibus salesnei*)	Bilabiate		Rounded	15.5	Haxaire and Salesne (2016), de Kok 574 (in herb.)
“*neriifolia*” subclade	*Oxera sororia*				Rounded	13.5	
“*oblongifolia*” subclade	*Oxera crassifolia*				Rounded	18.1	
“*oblongifolia*” subclade	*Oxera glandulosa*				Rounded	11.4	
“*oblongifolia*” subclade	*Oxera oblongifolia*		Bilabiate	Sturnidae (*Aplonis striatus*)	Rounded	14.6	de Kok 568 (in herb.)
“*oblongifolia*” subclade	*Oxera oreophila*	Lepidoptera	Bilabiate		Rounded	11.9	de Kok (1997)
*Oxera* *morierei* lineage	*Oxera morierei*	Honey eater (*Glycifohia undulata*)	Gullet		Rounded	16.2	de Kok (1997)
“*pulchella*” subclade	*Oxera aff. balansae*				Rounded	12.2	
“*pulchella*” subclade	*Oxera balansae*	Birds	Gullet	Sturnidae (*Aplonis striatus*)	Rounded	8.4	de Kok (1997), Desmoulins (pers. obs.)
“*pulchella*” subclade	*Oxera brevicalyx*	Honey eater (*Glycifohia undulata*)	Gullet	Sturnidae (*Aplonis striatus*)	Rounded	7.9	de Kok (1997), Desmoulins (pers. obs.)
“*pulchella*” subclade	*Oxera pulchella*	Honey eater (*Myzolema caledonica*), Zosteropidae *(Zosterops* sp.)	Gullet	Sturnidae (*Aplonis striatus*)	Rounded	11.3	Desmoulins (pers. obs.), Fleurot (pers. obs.)
“*robusta*” subclade	*Oxera coriacea*	Honey eater (*Glycifohia undulata*)	Gullet		Rounded	40.8	de Kok (1997)
“*robusta*” subclade	*Oxera coronata*				Rounded	29.9	
“*robusta*” subclade	*Oxera longifolia*	Honey eater (*Lichmera incana*)	Gullet		Rounded	37.2	de Kok (1997)
“*robusta*” subclade	*Oxera palmatinervia*	Honey eater (*Glycifohia undulata*, *Philemon diemenensis*)	Gullet	Columbidae (*Drepanoptila holosericea*, *Ducula Goliath*)	Rounded	34.0	de Kok (1997), Bachy (pers. obs.), Desmoulins (pers. obs.)
“*robusta*” subclade	*Oxera robusta*				Rounded	35.7	
“*subverticillata*” subclade	*Oxera aureocalyx* sp.nov.ined.				Paddle	40.7	
“*subverticillata*” subclade	*Oxera merytifolia*				Paddle	32.6	
“*subverticillata*” subclade	*Oxera subverticillata*	Honey eater (*Philemon diemenensis*)	Gullet		Paddle	30.9	de Kok (1997)
“*subverticillata*” subclade	*Oxera tiwaeana* sp.nov.ined.				Paddle	31.9	
“*sulfurea*” subclade	*Oxera gmelinoides*				Rounded	13.8	
“*sulfurea*” subclade	*Oxera microcalyx*	Honey eater (*Glycifohia undulata*), Zosteropidae (*Zosterops xanthochroa*)	Gullet		Rounded	13.5	de Kok (1997)
“*sulfurea*” subclade	*Oxera ouameniensis* sp.nov.ined.				Rounded	12.7	
“*sulfurea*” subclade	*Oxera pancheri*				Rounded	13.5	
“*sulfurea*” subclade	*Oxera rugosa*				Rounded	18.4	
“*sulfurea*” subclade	*Oxera sulfurea*	Honey eater (*Lichmera incana*, *Myzomela caledonica*, *Glycifohia undulata*)	Gullet	Sturnidae (*Aplonis striatus*)	Rounded	11.7	de Kok (1997), Desmoulins (pers. obs.), Gâteblé (pers. obs.)

These pollinator preferences could potentially be drivers of evolution of reproductive isolation within *Oxera*. Honey-eaters (Meliphagidae) and white-eyes (Zosteropidae) have been observed visiting *Oxera* species displaying showy and curved flowers, profusely producing nectar, and bearing arched anthers typically extending out of the corolla (Table 1). Long-tubular white and/or green bilabiate flowers, sometimes releasing a strong fragrance, have been observed to be visited by butterflies and moths (Pieridae and Sphingidae; Table 1; de Kok 1997; Haxaire and Salesne 2016). Despite the lack of recorded visitor observation, the flower attributes of the Australasian *O. splendida*, which bears large, flared, white flowers that open nocturnally and release a strong fragrance, are very similar to that of *Fagraea*, renowned for its bat-pollination (Momose et al. 1998).

Fourth, the large variety of fruit morphology exhibited by *Oxera* suggests different dispersal agents, although these mechanisms have not been fully identified in the field (Table 1). We assume that the different behaviors and flight abilities of animal dispersers would have affected the spatial scale of gene flow, and thus produced different rates of genetic divergence between *Oxera* lineages, due to varied dispersal abilities and habitat selection of dispersers (Givnish 2010, Theim et al. 2014). The largest white fruits encountered in Australasia are consumed by bats and cassowaries (Richards 1990; Hutson et al. 1992; Cooper and Cooper 2004). Other large colored fruits (ca. $>$2 cm) could be exclusively eaten by imperial pigeons (*Ducula*, Columbidae), which is the only bird able to swallow and effectively disperse such large fruits (Meehan et al. 2002; Carpenter et al. 2003; Cibois et al. 2017; P. Bachy pers. comm. for *O. palmatinervia*). It is noteworthy that the sedentary behavior of pigeons confines them mostly to forest understories (Colombo 2008; Wotton and Kelly 2012). Smaller fruits (ca. $<$2 cm) are consumed by more diminutive birds such as Sturnidae (Table 1; Tassin et al. 2010), which probably move over short distances, but are able to shift between different vegetation types (Barré et al. 2006). Finally, large grey or brown fruits having armed appendages are considered to be consumed by large geckos (*Rhacodactylus*, *Mniarogekko*, and *Correlophus* genera) because they are not discouraged by the fruits’ repelling structures (Whitaker and Bauer, pers. comm.). Large geckos also have limited spatial movements (Clark et al. 2009), which may have constrained gene flow in *Oxera*.

Given the large diversity of morphological traits, ecological attributes, and geographical ranges exhibited by *Oxera*, we suspect that species diversification within subclades of this genus has been driven by several ecological and biological factors acting at different time periods, which is in a leapfrog pattern (as originally defined by Chase and Palmer 1997). Available information on species coexistence within similar regions or habitats may also help test whether sympatric speciation has occurred in relation to different pollination and dispersal niches. Indeed, such a scenario suggests that sister species with distinct biotic niches should co-occur either in the same region or habitat. Sister species divergence based only on ecological preference or geographic range would otherwise suggest that speciation mainly occurred through parapatric or allopatric speciation, respectively. We thus posit that phylogenetic patterns of species ranges and niches bear the signature of both selective and neutral forces as engines of clade diversification. Here, we report the first detailed phylogenetic reconstruction of this exceptional radiation of Lamiaceae, the genus *Oxera*, within the New Caledonian biodiversity hotspot, and attempt to identify the major factors that have shaped its current species diversity. We used a combination of phylogenetics, molecular dating, and comparative analyses of species diversification and niche evolution in order to 1) reconstruct the tempo and rate of species diversification in *Oxera*, 2) extricate and identify the different drivers of its diversification, and 3) appraise their respective effects. This study specifically asks a pivotal question: Is *Oxera* the single clear adaptive radiation in New Caledonia?

## Materials and Methods

### Species Sampling and Molecular Data Sets

In order to both establish species relationships and to estimate divergence times within the genus *Oxera,* the focal ingroup was composed of the 38 recognized species according to the most recent systematic investigations in New Caledonia (Gâteblé unpublished data, available at http://endemia.nc/flore/fiche588), and taxonomical revisions by de Kok and Mabberley (1999a, 1999b) for Australasia and Pacific. We added to the data set the sister lineage *Hosea*, as well as 10 species belonging to the closely related *Clerodendrum* clade identified in Yuan et al. (2010). Three more divergent species of Lamiaceae were used as external outgroups (i.e., *Ajuga chamaepitys*, *Teucrium pyrenaicum*, *Rotheca* sp. Wen 9487; Supplementary Appendix S1a; available on Dryad at https://doi.org/10.5061/dryad.mm1ft40). A total of 51 species were used in this sampling, which we refer to as the *Oxera* data set, including 22 species with previously published sequences (downloaded from GenBank), and 29 species with new sequences generated for this study. This molecular data set consisted of the 12 DNA loci sequenced in Barrabé et al. (2015). This allowed us to improve 1) the phylogenetic resolution and 2) branch length estimates relative to Barrabé et al. (2015). All new DNA sequences were generated using the DNA extraction, amplification, and sequencing protocols described in Barrabé et al. (2015). All accessions used in this study are listed in the Supplementary Appendix S1a available on Dryad.

For divergence time estimation, we also used two additional alignments composed of four DNA plastid regions (*matK*, *rps16*, *trnL-F,* and *trnL-trnF*; Supplementary Appendix S2a available on Dryad) from two data sets, with taxon sampling that spanned the Lamiales and Lamiaceae. The Lamiales data set included: 1) a subset of the *Oxera* data set (i.e., five species; Supplementary Appendix S1c available on Dryad); and 2) 177 representatives of various lineages within Lamiales, whose sequences were downloaded from GenBank (Supplementary Appendix S1c available on Dryad). The Lamiaceae data set was thus composed of 1) the aforementioned *Oxera* subset and 2) 59 taxa representing all major Lamiaceae lineages (downloaded likewise from GenBank; Supplementary Appendix S1b available on Dryad). This large-scale sampling allowed us to incorporate Lamiales and Lamiaceae fossils as calibration points (see below).

All DNA alignment matrices used in this study are available on Dryad.

### Phylogenetic Inference

For the three molecular data sets, phylogenetic inferences were conducted using Bayesian Markov Chain Monte Carlo (MCMC) as implemented in MrBayes v3.2.1 (Ronquist et al. 2012) based on single-locus and concatenated data sets. Best-fit nucleotide sequence evolution models were identified using jModelTest (Posada 2008) based on the Akaike criterion (more details on settings are provided in Supplementary Appendix S2a available on Dryad). The combined data sets were partitioned to allow each locus to possess specific model parameters (Nylander et al. 2004). The methodological approach of the Bayesian MCMC analyses followed that described in Barrabé et al. (2015) and the details of parameter settings are provided in Supplementary Appendix S2b available on Dryad. All analyses were run on the Cyber Infrastructure for Phylogenetic Research cluster (CIPRES; http://www.phylo.org/; last accessed 18 October 2017). The post-burn-in trees resulting from the MCMC stationary phase were used to construct a majority-rule consensus tree and calculate Bayesian posterior probabilities (PPs); clades were considered well-supported when PP values were $\ge$0.95). The three resulting consensus trees are available in TreeBASE (ID 23353) and Dryad.

In order to check for phylogenetic incongruence between nuclear and plastid loci, we also ran two phylogenetic reconstructions separately for all six concatenated nuclear regions and all six concatenated plastid regions, using the same MrBayes parameter settings and models of nucleotide evolution as above. These two analyses were only applied to the *Oxera* data set. We then assessed whether there was any supported (PP $\ge$0.95) incongruence between two trees.

### Fossil Dating and Divergence Time Estimation

We performed a two-step approach to estimate the temporal evolution of *Oxera.* This allowed us to test the impact of different dating calibrations and taxonomic sampling on divergence time estimates. In the first step, we performed a fossil calibration on the Lamiales and Lamiaceae data sets using fossils described in a recent Lamiaceae molecular dating study (Yao et al. 2016). We incorporated three Lamiaceae (*Melissa*, *Ocimum*, and *Stachys*) and three Lamiales fossils (Bignoniaceae, Acanthaceae, and Oleaceae) for dating the phylogeny inferred with the Lamiales data set, and two Lamiaceae fossils (*Melissa* and *Ocimum*) for dating the phylogeny inferred with the Lamiaceae data set (for further details on the design and parameters of these calibrations, see Supplementary Appendix S2c, available on Dryad and explanations provided inYao et al. 2016). For root calibrations we used divergence times estimated in Magallón et al. (2015): 1) the divergence between Lamiales and Gentianales—Solanales for the Lamiales data set and 2) the divergence between Plocospermataceae and other Lamiales for the Lamiaceae data set. In the second step, the divergence time estimate between *Rotheca* (sp. Wen 9487) and other Lamiaceae recovered during the first step (i.e., the dating of the Lamiaceae data set) was used as a secondary root calibration point (see Results section) for the *Oxera* data set.

For the three molecular data sets, molecular divergence times were estimated using the Bayesian MCMC approach implemented in BEAST v.1.8.0 (Drummond and Rambaut 2007). DNA regions were combined and partitions were set as in the above Bayesian MCMC analyses (Supplementary Appendix S2c available on Dryad). An uncorrelated relaxed molecular clock model was selected following a lognormal distribution, and the Birth–Death process implemented for the tree prior. Other details on analyses, calibration and parameter settings are provided in Supplementary Appendix S2c available on Dryad. The post burn-in trees were summarized, and Bayesian PPs, median height (= age estimate), and the 95% highest posterior density heights interval of each node (95% HPD) assessed, using a Maximum Clade Credibility target tree (named as MCCT tree for the *Oxera* data set) in Treeannotator v.1.8.0 (Drummond and Rambaut 2007). For the *Oxera* data set a subset of 100 dated trees (RD trees hereafter) were also randomly sampled from the stationary phase to integrate phylogenetic uncertainty in some of the following analyses (see below). All divergence time analyses were conducted using CIPRES. The three resulting Maximum Clade Credibility trees are available in TreeBASE (ID 23353) and Dryad.

### Comparative Analyses of Species Diversification

To obtain an overall view of the *Oxera* diversification, we first generated a lineage through time diagram, based on a pruned version of the MCCT tree including only *Oxera* species and a single sample per species (pMCCT tree), and also on the RD trees (pruned as in the pMCCT tree). We also calculated net diversification rates, following the conservative approaches implemented in Pillon (2012) and Barrabé et al. (2014), to allow comparisons with other oceanic insular plant radiations, using median crown ages and their 95% HPD estimated from the molecular dating, and two extreme values for relative extinction (null and equal to 0.9). Net diversification rates were likewise estimated per unit of area and log(area).

To assess more precisely the rate and tempo of species diversification in *Oxera* through time, we carried out diversification analyses with maximum likelihood (ML) and Bayesian modeling approaches. The ML analyses were conducted in the TreePar R package (Stadler 2011), using an optimization algorithm to infer past rates of speciation and extinction and their temporal variation. We fitted various diversification models: a pure birth model, a constant birth–death model, several more complex models allowing one to several rate shifts through time (up to 10 shifts), and finally several diversity-dependent models. All models were fitted on the pMCCT tree (see Supplementary Appendix S2d, available on Dryad for further details on parameter settings) and on the RD trees to account for phylogenetic uncertainty. The best-fit diversification model was identified using the corrected Akaike criterion, and a likelihood-ratio test was used for comparing nested pure birth, birth–death, and rate shift models.

The Bayesian diversification analyses were computed using BAMM (Rabosky et al. 2014). This allows modelling complex dynamics of speciation and extinction on phylogenetic trees using a reversible jump MCMC algorithm. We pruned the MCCT tree in the same manner as the pMCCT tree, but retained species belonging to the *Clerodendrum* clade to assess early variation in *Oxera* diversification rates. The sampling fraction was completed with an estimate of species richness, especially for the *Clerodendrum* clade (retrieved from the Kew Checklist website: http://wcsp.science.kew.org; last accessed 18 October 2017). As we expected two rate shifts to have taken place during the evolution of *Oxera* and its relatives (considering the large sizes of the genera *Clerodendrum* and *Oxera* as compared with the other small genera), the prior on the number of diversification shifts was set to 2. All other priors were estimated in the BAMMtools R package (Rabosky et al. 2014), with the default settings used for all other parameters. The MCMC was run for $50 \times 10^{6}$ generations and sampled every 10,000 generations. The analysis was conducted with three independent Markov chains. The first 10% generations of each run were discarded after checking for chain convergence and adequate MCMC sampling. The remaining generations were summarized to generate the 95% credibility interval for rate shift configurations, the marginal shift probability tree (where branch lengths are proportional to the probability that a shift occurred on a given branch), the best rate shift configuration with the highest maximum a posteriori probability, the phylorate plot (showing variation of mean diversification rates with a colored gradient) and finally the rates-through time plots (only built for the *Oxera* focal group).

### Biological, Ecological, and Geographic Range Data sets

To identify and disentangle suspected drivers of species diversification in *Oxera*, we built a data set by compiling data on five major life traits: 1) flower morphology, 2) fruit morphology, 3) life forms, 4) geographical occurrences, and 5) environmental preferences. The flower and fruit morphology (i.e., organ dimensions, colors, shapes, and textures) were compiled from herbarium and fieldwork observations on organ dimensions, colors, shapes, and textures. Those traits were coded as 13 discrete and three continuous characters for flower morphology, and three discrete and three continuous traits for fruits (Supplementary Appendix S3a, d, and e available on Dryad). The life form data set was composed of a single discrete trait corresponding to the four life forms usually ascribed to *Oxera*, namely monocaulous, robust liana, slender liana, and shrub/small tree (Supplementary Appendix S3a and b available on Dryad). Note that no functional or physiological traits related to light requirements or nutrient use strategy were available for *Oxera*.

The geographical distributions of New Caledonian species were retrieved from the databases of the herbaria of Nouméa (NOU, VIROT), the Museum national d’Histoire naturelle of Paris (P, SONNERAT), the University of Zurich (Z), and additional field observations. The geographical coordinates of herbarium specimen records were then databased, error-corrected for distribution, and finally incorporated into a Geographical Information System (GIS) under QGIS 2.8.1. For Australasian and Pacific species distributions were assessed from de Kok and Mabberley (1999b) and manually added into the same GIS layer.

The environmental data set was composed of three variables that best explain plant distributions across New Caledonia (Jaffré 1993; Morat 1993; Supplementary Appendix S3a and c available on Dryad): geological substrates, vegetation types, and elevation. Species’ rock types were coded as occurring on ultramafic rocks, on volcano-sedimentary and metamorphic rocks, or ubiquists. These geological attributes were determined by coupling geographical occurrences with a layer of geological substrate provided by the Direction de l’Industrie, des Mines et de l’Energie de la Nouvelle-Calédonie (New Caledonia). Species’ vegetation types were coded as occurring in wet forests, in sclerophyllous vegetation types (i.e., dry forests and/or maquis vegetation), and ubiquists, based on herbarium and field observations (Gâteblé, pers. obs.). Species’ elevation ranges were extracted from the GIS layer generated by a digital elevation model (resolution of 10 m) provided by the Direction des Infrastructures de la Topographie et des Transports Terrestres (New Caledonia), and then averaged across geographical occurrences of each species.

To identify ecological syndromes among *Oxera* species, we performed multivariate analyses on the environmental, flower, and fruit data sets. As we used both continuous and discrete traits, we conducted the PCA analyses by using the Hill and Smith (1976) principal component method under the ade4 R package (Chessel et al. 2004). This analysis allowed clustering species among pollinator syndromes, dispersal syndromes, and habitat preferences, which we subsequently treated as new discrete traits according to the species clustering (i.e., the niche data sets; Supplementary Appendix S3f available on Dryad). We posit that the ecological syndrome of a species reflects the ecological niche it occupies (Johnson 2010, Pauw 2013). No data on species growth and architecture were available, we therefore, could not evaluate life forms in a similar manner; this trait was treated as a discrete variable without any step of ordination analysis.

### Comparative Analyses of Niche Evolution

To assess the mode and tempo of evolution of each niche during the *Oxera* radiation (i.e., of pollination and dispersal syndromes or habitat preferences), we measured their respective amount of phylogenetic signal by estimating $\kappa$ and $\lambda$ Pagel statistics (Pagel 1999). Values of $\kappa$ and $\lambda$ close to 1 indicate a strong phylogenetic signal and gradual trait evolution (i.e., according to a model of Brownian motion), and deviation from this expectation provides insight into temporal patterns of trait evolution. Values of $\kappa$ close to 0 depicts punctual evolution, where trait divergence occurs independently from branch lengths in the phylogeny. Values of $\lambda$ close to 0 reveal a low phylogenetic signal, where most trait change occurred late in evolutionary history and closely related species thus share very little trait similarity. For the habitat, pollination, and dispersal niches $\kappa$ and $\lambda$ statistics were estimated using the “fitContinuous” and “phylosig” functions of the geiger and phytools R packages, respectively (Harmon et al. 2008; Revell 2012). Estimations were performed on the RD trees, and by averaging estimated values for each of the first n axes of each PCA explaining 90% of the cumulative variance. For life form, each statistic was simply averaged through the RD trees using the “fitdiscrete” function (geiger) and the single discrete trait.

To reconstruct the evolutionary history of habitat preference, pollination, dispersal niches, and life form, we conducted ancestral state reconstruction analyses. We first assessed ancestral niche states using the “ace” function in the ape R package (Paradis et al. 2004) applied to the pMCCT tree, and the niche and life form data sets. We fitted three ML evolution models: the “ER” (with equal state transition rates), “SYM” (symmetrical), and “ARD” (with all rates unequal) models, identifying the best-fitting model using comparisons performed on the corrected Akaike criterion, and likelihood ratio test. We then estimated their respective absolute shift number and timing by performing stochastic character mappings (Huelsenbeck et al. 2003), using the “make.simmap” function in phytools. We launched 500 simulations using an estimated prior distribution on the root node, the best-fit ML model recovered in the previous analyses and applied first to the pMCCT tree, and then to the RD trees to obtain most likely envelopes of past shift number for habitat preference, pollination, dispersal niches and life-forms. For 11 of the 100 RD trees, we encountered intractable optimization issues when using the “make.simmap” function. We subsequently removed the 11 trees from these analyses and used only 89 RD trees. Absolute shift timings were extracted using a custom R-script from all simulations and summarized by plotting the median number of state changes in different time bins of a 0.5 Myr time-grid.

To investigate whether a particular niche state (pollination, dispersal, or habitat) or life-form would have affected clade diversification rates of *Oxera,* we conducted multi-state trait-based analyses that estimated simultaneously trait-dependent speciation, extinction, and transition rates on a phylogeny and character distribution (Fitzjohn et al. 2009). These were performed using the “make.musse” function under the diversitree R package (Fitzjohn et al. 2009), applied to the pMCCT tree, and the niche and life form data sets. We first fitted 17 models from a null model (rates independent of trait states) to a full model (all rate components dependent of trait states; Supplementary Appendix S4 available on Dryad), conserving the best-fitting using the corrected Akaike criterion. We performed subsequent Bayesian analyses through a MCMC, run for 10,000 generations, seeding them with rate estimates recovered from the previous ML analyses as priors, and discarding the first 1000 generations as burn-in. PP distributions of all parameters were summarized using the diversitree R-package. We also fitted the best model and compared it to the second best model across all RD trees to check that model selection was robust to phylogenetic uncertainty.

### Geographical Evolution versus Habitat Evolution

To investigate the relative roles of geographical and environmental divergence during the diversification of *Oxera*, we computed age-range correlations (Fitzpatrick and Turelli 2006; Warren et al. 2008) where metrics of range overlaps, range asymmetries, and habitat distances computed between species pairs are compared with their estimated time of divergence (from the phylogeny). These metrics have been recurrently used to diagnose different speciation and post-speciation scenarios (Anacker and Strauss 2014; Grossenbacher et al. 2014).

Species geographical ranges were retrieved from the geographical data set by creating for each species a single convex polygon enclosing its locations using the “convex hull” tool under QGIS. For narrow endemic species, with one or two occurrences, we applied a buffer of 0.5 km around each location, and used a range of 3.141592 $\times$ 0.5$^{2}$ km$^{2}$ for the former, and of 6.283184 $\times$ 0.5$^{2}$ km$^{2}$ for the latter. From these distribution data, range overlaps, range asymmetries, and habitat distances metrics were computed between all possible pairs of species. Range overlap was defined as the area occupied by two species divided by the area of the more narrowly ranging species, ranging from 0 (no co-occurrence) to 1 (complete co-occurrence). Range asymmetry was calculated as the area of the wider-ranging species divided by that of the smaller (ranging from 1 to infinity). The habitat similarity between two species was assessed by calculating their Euclidian distance (habitat distance) based on the eigenvector values of the first $n$ axes explaining 90% of the cumulate variance retrieved from the environmental PCA (see above). These geographical variables (areas of all *Oxera* species, range asymmetries and range overlaps of all *Oxera* species pairs) are available on Dryad.

Age-range correlation analyses were performed using an extended version of the “age.range.correlation” function of the phyloclim R package (Heibl and Calenge 2013; https://github.com/danlwarren/arc-extensions/blob/master/age.range.correlation.2.R; last accessed 18 October 2017), applied to our pMCCT and the RD trees. We launched 1000 iterations for the Monte Carlo resampling procedure to create the null hypothesis of no relationship between phylogenetic relatedness and range overlap, range asymmetry, or habitat distance, and tested it. We calculated linear regressions, whose slopes and intercepts indicate speciation mode (allopatric vs. sympatric) or habitat evolution (conservatism vs. divergence) through time. For each metric a “super-p-value” was calculated, corresponding to the tree proportion across the RD trees for which the linear regression was significant. We also assessed the frequency of range overlaps across all random species pairs and across all sister species pairs using a kernel density plot.

Finally, we estimated the respective importance of geographical versus habitat divergence and whether local co-existence is possible between closely related *Oxera* species. To do so, we superimposed species geographical overlaps to their habitat preferences (summarized into diagrams combining information on their geological, elevation, and vegetation attributes) within each *Oxera* subclade (as delimited in Barrabé et al. 2015) to determine whether closely related species could possibly occur in sympatry.

## Results

### Phylogenetic Inference

**Figure 2.**
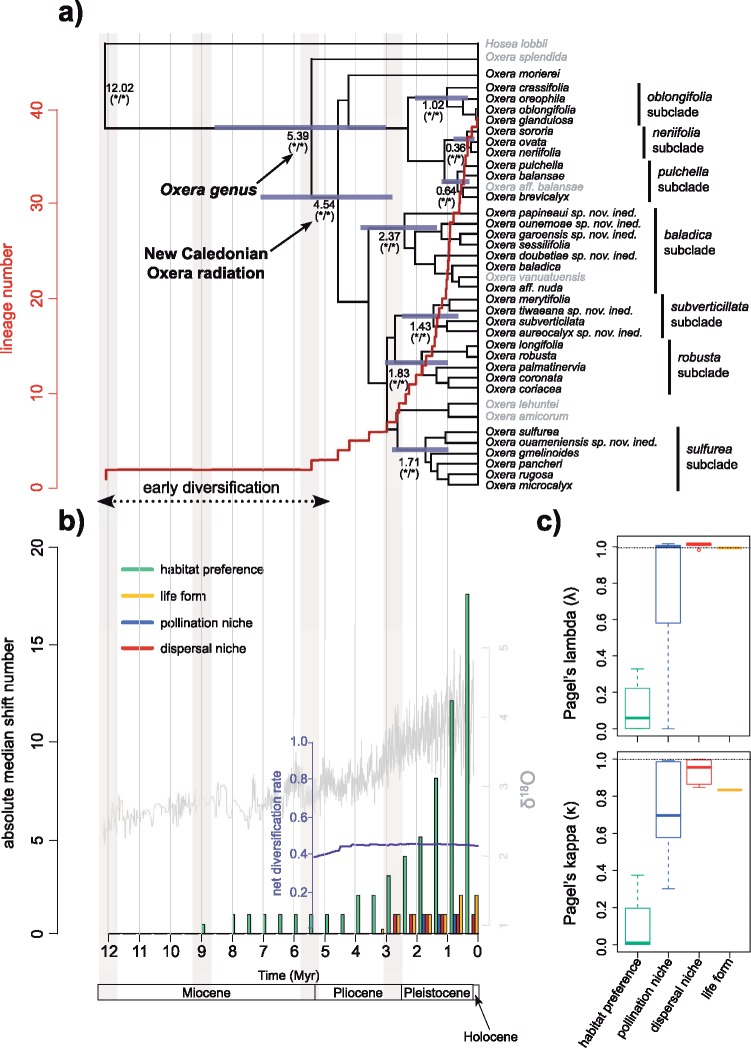
Evolutionary history of *Oxera* in New Caledonia, and of its habitat preferences, pollination and dispersal niches, and life forms. a) BEAST chronogram of the *Oxera* data set with superimposed lineage through time plot. Median age estimates and blue node bars (corresponding to the 95% highest posterior densities) are indicated for each node of interest. Bayesian PP from the BEAST analyses (on the right) and MCMC Bayesian analyses (on the left) are indicated with an asterisk for each node of interest when $>$0.95, and with a hyphen when $<$0.95. Vertical light grey rectangles indicate periods of intense weathering as inferred from Chevillotte et al. (2006). b) Absolute shift timings of the three niches and life forms inferred from stochastic character mappings, with global delta $^{18}$O variation through time (in grey; from Zachos et al. 2001) and net diversification rate variation through time of *Oxera*, as inferred from BAMM analyses (in dark blue), superimposed. c) Phylogenetic signal for habitat preferences, pollination and dispersal niches, and life forms inferred from estimations of the $\kappa$ and $\lambda$ Pagel statistics.

The Bayesian MCMC analyses of the three species sampling (Lamiales, Lamiaceae, and *Oxera* data sets) recovered the same following robust phylogenetic relationships (Supplementary Appendices S5–S7 available on Dryad). The sister genera *Hosea* and *Oxera* formed a well-supported clade that was sister to the *Clerodendrum* clade (PP $= 1$). Analyses based on the *Oxera* data set provided more resolution and detail on the internal *Oxera* relationships, which are congruent with the ones established in Barrabé et al. (2015) (Supplementary Appendix S5 available on Dryad). The Australasian *O. splendida* was placed as sister to the New Caledonian radiation (PP $= 0.72$), which included all New Caledonian and nested Pacific subclades. We retrieved seven well-supported New Caledonian subclades (PP $= 1$; Fig. 2a), namely the *oblongifolia*, *neriifolia, pulchella*, *baladica, subverticillata*, *robusta*, and *sulfurea* subclades.

A few supported incongruences (PP $\ge 0.95$) were observed between the phylogenetic trees reconstructed separately from the six nuclear loci and from the six plastid loci (Supplementary Appendix S8 available on Dryad), but these incongruencies were all located near the tips (i.e., between close species). This discordance did not affect the robustness of the seven New Caledonian subclades previously recovered. These minor incongruences underscore the importance of accounting for phylogenetic uncertainty by using the 100 RD trees in most of our evolution and geographical analyses (see Material and Methods section).

### Molecular Dating

The three molecular BEAST dating analyses provided congruent divergence times for *Oxera* and relatives (Fig. 2a, Supplementary Appendices S9–S11 available on Dryad), which can thus be considered as a reliable appraisal of colonization dates and tempo of species diversification. *Oxera* likely emerged during the late Miocene, and the New Caledonian radiation *sensu stricto* is estimated to have initiated during the early Pliocene. The median divergence time estimates for the stem node of *Oxera* were 11.46 Ma (95% HPD = 5.65–19.68), 11.2 Ma (95% HPD $=$ 5.61–19.06), and 12.02 Ma (95% HPD $=$ 6.21–19.16), as recovered with the Lamiales, Lamiaceae, and *Oxera* data sets, respectively (Fig. 2a, Supplementary Appendices S9–S11 available on Dryad). For the crown node, age estimates were 6.34 Ma (95% HPD $=$ 2.89–11.48), 6.28 Ma (95% HPD $=$ 3.07–10.73), and 5.39 Ma (95% HPD $=$ 2.97–8.42), respectively. The *Oxera* data set provided an estimate of 4.54 Ma (95% HPD $=$ 2.7–6.92) for the crown node of the New Caledonian radiation (Fig. 2a).

### Oxera Diversification

The lineage through time plot highlighted a gradual and exponential increase of the number of *Oxera* lineages through time (Fig. 2a). The net conservative diversification rates are summarized in Tables 2 and 3. For the New Caledonian radiation, these rates were 0.2 species/Myr for an extinction of 0.9 and 1.04 species/Myr for a null extinction. With respect to per-unit-area and per-unit-log(area) they were estimated between $1.1 \times 10^{-5}$ and $5.6 \times 10^{-5}$ species/Myr/km$^{2}$, and between 0.02 and 0.106 species/Myr/log(km$^{2})$, respectively. The BAMM analyses inferred two early instances of changes in the diversification rate. The phylorate plot showed an increase in mean diversification rates at the crown node of *Oxera* and the second deepest node of the *Clerodendrum* clade, whereas *Hosea* demonstrated a decrease in these rates (Supplementary Appendix S12a available on Dryad). Five of the six most probable 95% credible rate shift configurations (with a cumulative PP of 0.378; Supplementary Appendix S13 available on Dryad) showed significant rate shifts mainly located on the two consecutive branches leading to the New Caledonian *Oxera* radiation, and those leading to most of the *Clerodendrum* clade (Supplementary Appendix S13 available on Dryad). Both shifts were depicted in the marginal shift probability tree, where these latter branches were significantly longer than most others, indicating a high probability that these shifts occurred on their respective branches (Supplementary Appendix S12b available on Dryad). The *Oxera* rates through time plots highlighted that speciation rates remained constant (with a mean value of ca. 0.71 species/Myr). Extinction rates decreased slightly at the radiation onset but remained constant after (mean value of ca. 0.31 species/Myr). The resulting net diversification rates increased early in the radiation onset and later remained quite constant (mean value of ca. 0.4 species/Myr; Fig. 2b, Supplementary Appendix S12c available on Dryad). The ML diversification analyses selected the pure birth model as best-fitting our data for the genus *Oxera,* with a net diversification rate of 0.575 species/Myr (Supplementary Appendix S14 available on Dryad). Accordingly, the pure birth model was the best selected model on 93% of the RD trees.

**Table 2.** Comparison of net diversification rates of *Oxera* with other New Caledonian plant lineages/radiations

Family	Radiation	Stem age (Ma)	Crown age (Ma)	Number of New Caledonian species retrieved from Munzinger et al. (2016)	Species number restricted to sclerophyll vegetations	Species number able to growth in sclerophyll vegetations	Proportion of species strictly growing in sclerophyll vegetations (%)	Proportion of species able to grow in sclerophyll vegetations	Net diversification rate (based on the equation of Magallón and Sanderson (2001)) for an extinction null, and using crown ages (in species/Myr)	Works where age estimates have been published
Sapotaceae	*Planchonella* clade III	18	15	3	1	2	33.3	66.7	0.03	Swenson et al. (2013)
Proteaceae	*Beauprea* clade I (“filipes” clade)	78.2	28.3	7	1	5	14.3	71.4	0.04	He et al. (2016)
Nothofagaceae	*Nothofagus*	26.1	16.4	5	0	0	0	0	0.06	Sauquet et al. (2012)
Sapotaceae	*Planchonella* clade I	15.3	12.05	4	0	3	0	75	0.06	Swenson et al. (2013)
Proteaceae	*Kermadecia - Sleumerodendron*	13.6	12.3	5	0	0		0	0.07	Sauquet et al. (2009)
Myrtaceae	*Metrosideros* clade B	21.25	11	5	1	3	20	60	0.08	Papadopulos et al. (2011)
Myrtaceae	*Metrosideros* clade A	16.25	13.6	7	4	7	57.1	100	0.09	Papadopulos et al. (2011)
Arecaceae	*Clinosperma—Cyphokentia*	21.5	10.5	6	0	0	0	0	0.1	Baker and Couvreur (2012)
Myrtaceae	*Metrosideros* clade C	29.1	8.25	5	2	4	40	80	0.11	Papadopulos et al. (2011)
Arecaceae	*Chambeyronia—Kentiopsis*	9	4.5	6	0	0	0	0	0.12	Baker and Couvreur (2012)
Sapotaceae	*Pichonia*	19	10.1	7	5	5	71.4	57.1	0.12	Swenson et al. (2013)
Sapotaceae	*Planchonella* clade II	33	17.4	18	7	7	38.9	38.9	0.13	Swenson et al. (2013)
Rubiaceae	*Margaritopsis*	8.47	5.21	4	1	4	25	100	0.13	Barrabé et al. (2014)
Rubiaceae	*Psychotria* clade NC1	17.06	4.78	4	4	4	100	100	0.15	Barrabé et al. (2014)
Rutaceae	*Oxanthera*	max 5.8	5.8	5	5	5	100	100	0.16	Pfeil and Crisp (2008)
Sapotaceae	*Pleioluma* clade I	10	4.9	5	0	2	0	40	0.19	Swenson et al. (2013)
Arecaceae	*Burretiokentia—Cyphophoenix*	11.5	7.5	9	0	0	0	0	0.2	Baker and Couvreur (2012)
Sapotaceae	*Pycnandra*	29.8	16.2	57	17	20	29.8	35.1	0.21	Swenson et al. (2013)
Araucariaceae	*Araucaria*	16	8	13	0	7	0	53.8	0.23	Kranitz et al. (2014)
Loganiaceae	*Geniostoma*	9	6.5	10	1	4	10	40	0.25	Foster et al. (2014)
Podocarpaceae	*Dacrydium*	5	3.05	5	0	3	0	60	0.3	Keppel et al. (2011)
Podocarpaceae	*Podocarpus*	6.9	4.2	8	2	2	25	25	0.33	Quiroga et al. (2016)
Sapotaceae	*Pleioluma* clade II	7.5	4.2	8	6	8	75	100	0.33	Swenson et al. (2013)
Anacardiaceae	*Euroschinus*	11.85	3.7	7	0	2	0	28.6	0.34	Weeks et al. (2014)
Ebenaceae	*Diospyros* clade III	9.1	7.2	25	8	16	32	64	0.35	Turner et al. (2013)
Pandanaceae	NC *Pandanus* subgen. *Lophostigma*	8.5	6.5	24	2	2	8.3	8.3	0.38	Gallaher et al. (2015)
Ericaceae	*Dracophyllum*	5.2	3.5	8	7	6	87.5	75	0.4	Wagstaff et al. (2010)
Rubiaceae	*Psychotria* clade NC2	7.63	6.9	78	32	40	41	51.3	0.53	Barrabé et al. (2014)
Lamiaceae	NC *Oxera*	5.39	4.54	33	5	11	15.2	29.7	0.62	This study
Rubiaceae	*Thiollierea*	6.5	3	13	13	13	100	100	0.62	Manns et al. (2012)

*Note*: In grey, sclerophyllous clades.

**Table 3.** Comparison of net diversification rates of *Oxera* with other rapid island plant lineages/radiations

Family	Radiation	Archipelago	Number of species	Area (km$^{2})$	Median crown age (95% HPD in Ma)		Net diversification rate and using crown ages (species/Myr)		Net diversification rate at a area unit (species/ Myr/km$^{2})$	Net diversification rate at a log(area) unit (species/Myr/ log(km$^{2}))$	Works where age estimates and species number have been published
							Extinction = 0	Extinction = 0.9			
Lamiaceae	New Caledonian *Oxera*	New Caledonia	33	18,600	2.7	6.92	0.41–1.04	0.2–0.51	0.000011–0.000056	0.02–0.106	This study
Rubiaceae	*Psychotria* clade NC2	New Caledonia	78	18,600	4.62	9.82	0.37–0.79	0.22–0.46	0.000012–0.000043	0.022–0.081	Barrabé et al. (2014)
Asteraceae	*Cheilorophus*	Macaronesia	20	10,372	5.98	15.35	0.15–0.39	0.07–0.17	0.000006–0.000037	0.007–0.042	Vitales et al. (2014)
Euphorbiaceae	*Euphorbia*	Hawaii	16	16,644	0.72	3.97	0.52–2.89	0.22–1.2	0.000013–0.000174	0.022–0.297	Yang et al. (2018)
Asteraceae	*Bidens*	Hawaii	19	16,644	1.3	3.1	0.73–1.73	0.32–0.75	0.000019–0.000104	0.032–0.178	Knope et al. (2012)
Asteraceae	*Echium*	Macaronesia	19	10,372	2.7	3.9	0.58–0.83	0.25–0.36	0.000024–0.00008	0.027–0.09	Knope et al. (2012)
Gesneriaceae	*Cyrtandra*	Hawaii	58	16,644	5.2		0.65	0.36	0.000021–0.000039	0.037–0.067	Clark et al. (2008, 2009)
Campanulaceae	Lobeliads	Hawaii	126	16,644	10.49	16.71	0.25–0.39	0.15–0.24	0.000009–0.000024	0.016–0.041	Givnish et al. (2009)
Crassulaceae	*Aeonium*	Macaronesia	63	10,372	13.25	17.15	0.2–0.26	0.11–0.15	0.000011–0.000025	0.012–0.028	Kim et al. (2008)
Plantaginaceae	*Veronica*	New Zealand	120	268,680	5.7		0.72	0.44	0.000002–0.000003	0.035–0.057	Ogutcen (2016), Wagstaff et al. (2002)

### Niche Ordination

The multivariate analyses allowed the separation of four, three, and five clearly distinct ecological syndromes from the species’ environmental, flower, and fruit data sets, respectively (Fig. 1c). The first four, nine, and six axes of the PCA analyses (explaining 90 % of the cumulate variance), respectively, were retained for subsequent comparative analyses. The three major habitats occupied by *Oxera* were forests on non-ultramafic rocks, forests on ultramafic rocks, sclerophyllous vegetation, and a fourth class was defined for ubiquist species. The three pollination syndromes characterized species with actinomorphic, gullet, and bilabiate floral morphs each adapted to bat, bird, moth/butterfly pollination, respectively. For dispersal syndromes the PCA axis markedly separated species with small fruits $\le2$ cm (on the left) from those with large fruits $>$2 cm (on the right; Fig. 1c). The five dispersal syndromes discriminated species with potato-like (consumed by cassowaries and bats), paddle-like (by Columbidae), small rounded (by Columbidae and/or small birds such as Sturnidae), large rounded (by Columbidae), and thorny fruit morphs (presumably by large geckos).

### Niche Evolution

Our estimates of both Pagel statistics showed distinct evolutionary patterns (Fig. 2c; Supplementary Appendix S15 available on Dryad). We found low values of $\lambda$, indicating low phylogenetic signal (i.e., a recent accelerated evolution), for the habitat preference (Fig. 2c), and high phylogenetic signal for the dispersal niche and life form, suggesting that these niche characteristics have diversified early. For the pollination niche, estimates of $\lambda$ were more spread out, with a lower 5% quantile of 0.2, a higher 95% quantile of 1.01, and a median value of 0.99; this also indicated a relatively high phylogenetic signal (Fig. 2c). Estimates of the $\kappa$ statistic were close to 0 for the habitat preference (indicating a punctual evolution), and close to 1 for the dispersal niche and life form, respectively (indicating more gradual evolution). For the pollination niche, $\kappa$ scaled between 0.35 (5% quantile) and 0.99 (95% quantile), with a median value of 0.69.

The best-fit evolutionary model identified in our ancestral state reconstruction analyses was the “ER” model for each niche/trait data set (Supplementary Appendix S16 available on Dryad). The reconstructed ancestral habitat preference of *Oxera* was ambiguous: forests on both ultramafic and non-ultramafic rocks were together reconstructed with a high likelihood (forested vegetation was recovered as ancestral for this node with cumulated likelihoods of both forest types of ca. 0.57). However, the ancestral pollination, dispersal, and life form were inferred with much less ambiguity; the *Oxera* ancestor was very likely a robust liana with gullet flowers producing small rounded fruits (Supplementary Appendix S17 available on Dryad). Analyses of stochastic character mapping allowed us to estimate that very few pollination, dispersal, and life form shifts occurred during the evolution of *Oxera* (up to two shifts per time bin of 0.5 Myr; Fig. 2b), and that most of these trait shifts occurred between 2 Ma and 0.5 Ma for pollination, and following 3 Ma and 3.5 Ma for dispersal and life form, respectively. Habitat shifts occurred between 9 Ma and present, but from 4 Ma onward their number increased markedly, finally reaching 17 shifts between 0.5 Ma and the present.

The best-fitting model of trait-dependent species diversification identified for habitats had speciation and transition rates all equal, a null extinction rate (Supplementary Appendix S4 available on Dryad), and no discernible effect of any habitat type. For the pollination syndromes, dispersal types and life forms, the best-fitting model was that with a null extinction, transition rates all equal, and a particular character state in which speciation rates were significantly different from other states (Supplementary Appendix S4 available on Dryad). These results were very robust to phylogenetic uncertainty, as 100% of the RD trees consistently yielded the same best models. Slender lianas had on average significantly higher speciation rates comparing to other life forms, actinomorphic flowers, and potato-like fruits had on average significantly lower speciation rates comparing to other floral and fruit types (Supplementary Appendix S18 available on Dryad).

### Geographical Evolution

**Figure 3.**
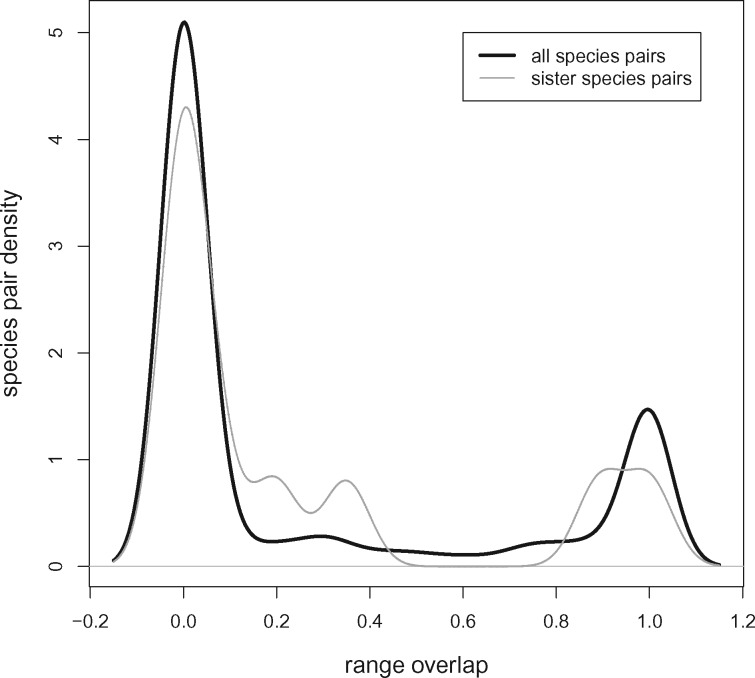
The Kernel density plot of range overlap through all possible species pairs (in black) and all possible sister species pairs (in grey) in New Caledonian *Oxera.*

The range overlap between all species pairs was generally low (mean value of 0.187, and a standard deviation of 0.357), suggesting a high level of allopatry between all species. The kernel density plot highlighted two range overlap frequency peaks, the highest located for a range overlap of 0 and the shortest to 1 (Fig. 3). Age range correlation analyses showed that the slope of range asymmetry through time was significantly positive for linear regressions (all $P$-values $<$0.05 across RD trees, Supplementary Appendix S19 available on Dryad). For range overlap and habitat distance, linear regression slopes with divergence times were negative in both cases but not significant.

**Figure 4.**
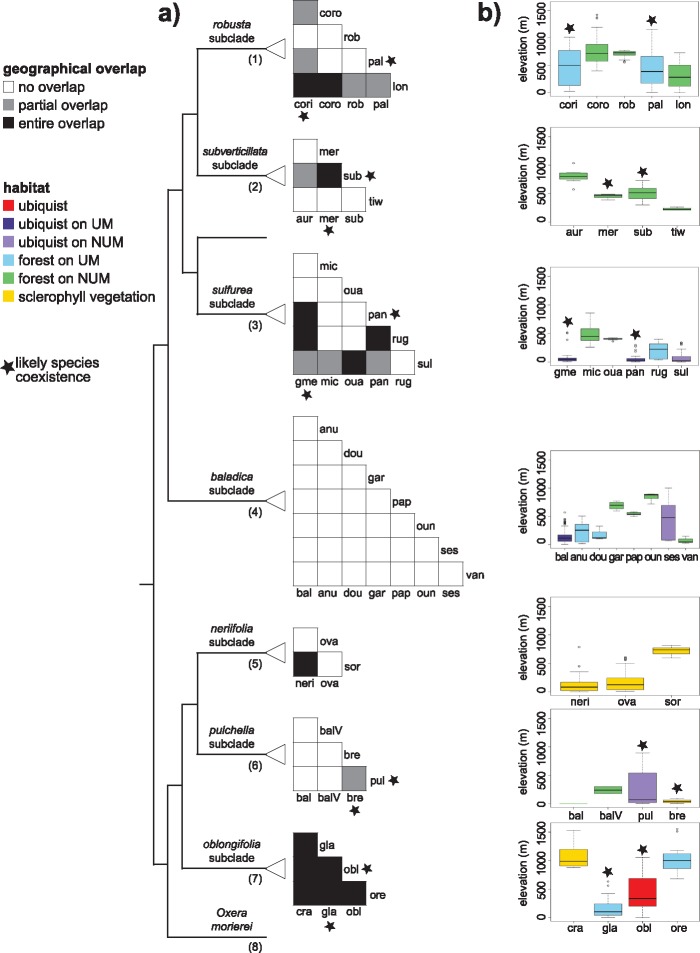
Species coexistence within *Oxera,* indicated by subclade, with stars indicating likely coexistence; mapped on the BEAST maximum credibility clade chronogram from the *Oxera* data set. a) Geographical overlap between all species of each subclade. b) Ecological ranges of all species indicated per subclade, plotted according to their altitudinal ranges and their habitat preferences (UM $=$ ultramafic soils, NUM $=$ non-ultramafic soils). Species names are indicated with the following abbreviations, listed according to subclade: *robusta* subclade (cori $=$*O. coriacea*, coro $=$*O. coronata*, lon $=$*O. longifolia*, pal $=$*O. palmatinervia*, rob $=$*O. robusta*), *subverticillata* subclade (aur $=$*O. aureocalyx* sp. nov. ined., mer $=$*O. merytifolia*, sub $=$*O. subverticillata*, tiw $=$*O. tiwaeana* sp. nov. ined.), *sulfurea* subclade (gme $=$*O. gmelinoides*, mic $=$*O. microcalyx*, oua $=$*O. ouameniensis* sp. nov. ined., pan $=$*O. pancheri*, rug $=$*O. rugosa*, sul $=$*O. sulfurea*), *baladica* subclade (bal $=$*O. baladica*, anu $=$$O$. aff. *nuda*, dou $=$*O. doubetiae* sp. nov. ined., gar $=$*O. garoensis* sp. nov. ined., oun $=$*O. ounemoae* sp. nov. ined., pap $=$*O. papineaui* sp. nov. ined., ses $=$*O. sessilifolia*, van $=$*O. vanuatuensis*), *neriifolia* subclade (neri $=$*O. neriifolia*, ova $=$*O. ovata*, sor $=$*O. sororia*), *pulchella* subclade (bal $=$*O. balansae*, balV $=$$O.$aff. *balansae*, bre $=$*O. brevicalyx*, pul $=$*O. pulchella*), and *oblongifolia* subclade (cra $=$*O. crassifolia*, gla $=$*O. glandulosa*, obl $=$*O. oblongifolia*, ore $=$*O. oreophila*).

Different *Oxera* subclades highlighted distinct patterns of geographical overlap (Fig. 4a and b). The *baladica* subclade was entirely and strictly allopatric since none of its species overlapped geographically. The *oblongifolia* subclade was completely sympatric as all its species overlapped. The other subclades (i.e., “*neriifolia*” “*pulchella*” “*robusta*” “*subverticillata*” and “*sulfurea*” subclades) exhibited more complex patterns (Fig. 4a). Overall, of the 96 possible pairs of species encountered within the seven New Caledonian subclades, we recorded 23 cases of partial or entire geographical overlap (Fig. 4a and b), but most of them involved species occurring in markedly distinct habitats. In fact, among all 96 species pairs, only five pairs involved geographically overlapping pairs of species growing in the same habitat, suggesting possible local co-existence (i.e., *O. oblongifolia* and *O. glandulosa, O. coriacea, and O. palmatinervia, O. merytifolia, and O. subverticillata, O. gmelinoides, and O. pancheri*). However, none of these species pairs have been observed co-occurring in the field (Gâteblé, pers. obs.).

## Discussion

### An Early Burst of Diversification

Our study shows that the genus *Oxera* is a quite recent and rapid plant radiation that initiated during the late Miocene, and that most of the New Caledonian subclades within *Oxera* began to diversify in the early Pliocene (Table 2, Fig. 2). It is noteworthy that this New Caledonian radiation occurred long after the emergence of Grande Terre about 37 Ma (Cluzel et al. 2012). Thus, the radiation of *Oxera* originated in the archipelago via long-distance or stepping-stone dispersal, but was not triggered by Gondwanan fragmentation. This continental dispersal to the New Caledonian archipelago was probably aided by the ancestral dispersal type (i.e., small rounded fleshy fruits; Supplementary Appendix S17 available on Dryad), which may be the most efficient dispersal mode in the group. Following this dispersal to New Caledonia, the successful establishment of ancestral *Oxera* may have been facilitated by the climbing habit of its ancestor (robust woody liana identified as the ancestral state; Supplementary Appendix S17 available on Dryad), which is believed to allow the occupation of a broad range of local environments (Gianoli 2004). The finding that the ancestral colonizer of extant New Caledonian *Oxera* was reconstructed as having a liana life-form with fleshy fruits makes it a quite unique case since the liana form is generally associated with wind dispersal (Givnish 2010), implying dispersal limitations over water, and that the liana niche in islands remains often empty.

Diversification analyses identified an early burst of diversification (BAMM analyses; Fig. 2a and b, Supplementary Appendix S12 available on Dryad), likely located between the divergence of the *Oxera* ingroup and its sister lineage *Hosea* (ca. 12 Ma) and the crown node of *Oxera* within New Caledonia (ca. 4.5 Ma). This late Miocene/early Pliocene burst of species diversification indicates that *Oxera* underwent an initial period of rapid cladogenesis, especially in New Caledonia, concordant with an initial decreasing of extinction rates; Figure 2b, Supplementary Appendix S12 available on Dryad. This coincides with the origination and early diversification of other major New Caledonian radiations (e.g., *Psychotria* clade NC2), and especially lineages typical of sclerophyllous vegetation types (e.g., *Thiollierea*; Table 2).

This period of intense species diversification is concordant with the global onset of Pliocene glaciations (Zachos et al. 2001) as well as the temperature declines and severe aridification recorded in the South Pacific during the Pliocene (Karas et al. 2011). It also coincides with the timing of intense soil morphogenesis and weathering which is estimated to have occurred around 5.5 Ma in New Caledonia (Fig. 2a and b; Chevillotte et al. 2006). Such climatic events have been suggested to have impacted the evolutionary history of many plant lineages by leading to the extinction of certain species (thus alleviating competitive effects), and the emergence of new biotas (e.g., more sclerophyllous and open vegetation), especially since the Miocene (Donoghue and Edwards 2014). It is important to note that some of these novel habitats exhibit a patchy spatial distribution, which has likely increased genetic divergence. This ostensibly has generated vacant niches, created ecological opportunities, increased spatial divergence and thus triggered speciation in many Australasian lineages, as highlighted in Australia for *Banksia, Eucalyptus*, and *Allocasuarina* (Crisp et al. 2004; Gallagher et al. 2003). The early rapid diversification of *Oxera* suggests that similar climatic and physical events fostered diversification within *Oxera* shortly after its arrival into New Caledonia.

### An Explosive Species Diversification, with Early Divergence in Biotic Niches

The rate of species diversification estimated for *Oxera* is exceptionally high, and represents the fastest known New Caledonian plant radiation, rivalled only by *Thiollierea* (Manns et al. 2012; Table 2). Estimated speciation rates within *Oxera* are comparable to the fastest island plant radiations documented in previous studies (e.g., *Bidens* in Hawaii or *Echium* in Macaronesia; Table 3). The detected early burst of diversification has been followed by a rather constant diversification, as inferred from BAMM and ML Treepar analyses even under weak divergence origination scenarios (Supplementary Appendices 12 and 14 available on Dryad); this may reflect the ongoing challenge within the evolutionary biology community to realistically model diversification processes, especially extinction (Stadler 2011; Morlon 2014). Nevertheless, it seems that since its origination *Oxera* has continued to diversify under constant speciation and rather null extinction, as exemplified by the exponential increase of species lineages through time (Fig. 2a).

Trait-dependent diversification models suggest that no specific adaptation has disproportionately favored speciation in *Oxera* (all biotic traits had equal speciation rates; Supplementary Appendices S4 and S18 available on Dryad), excepting perhaps the slender liana life form (acquired with the emergence of the *neriifolia*, *pulchella,* and *oblongifolia* subclades; see Table 4, Fig. 2a, Supplementary Appendix S18 available on Dryad), which is found in open sclerophyllous vegetation and exhibits high speciation rates probably because open habitats have increased in concert with Pliocene and Pleistocene cooling while remaining spatially fragmented (see below). However, the proliferation of species within *Oxera* might be related to the capacity of the genus to adopt different growth forms and floral and fruit morphologies, as hypothesized for angiosperms in general (Ricklefs and Renner 1994), probably in response to varied ecological opportunities available after its colonization of New Caledonia.

**Table 4.** Combinations of ancestral node states (or character state for the single-species *morierei* lineage) of life forms, pollination and dispersal niches for each *Oxera* subclade, inferred from ancestral state reconstruction analyses

Subclade/lineage	Life form	pollination niche	dispersal niche
*robusta* (1)	Robust liana	Gullet	Large rounded
*subverticillata* (2)	Robust liana	Gullet	Paddle
*sulfurea* (3)	Shrub	Gullet	Small rounded
*baladica* (4)	Monocaulous	Gullet	Paddle
*neriifolia* (5)	Slender liana	Bilabiate	Small rounded
*pulchella* (6)	Slender liana	Gullet	Small rounded
*oblongifolia* (7)	Slender liana	Bilabiate	Small rounded
*morierei* (8)	Slender liana	Gullet	Small rounded

Numbers in brackets refer to tree node numbers in Figure 4. Clades with similar combinations are highlighted in grey.

The early diversification of *Oxera* seems to have been mainly associated with niche shifts driven by pollination syndromes, dispersal strategies, and life-form variety (Fig. 2b). This suggests that the early diversification burst within the clade coincides with ecological diversification, particularly in regards to pollen vectors, dispersal agents, and light requirements. Although it is not possible to determine whether these niche shifts were directly involved in the process of lineage divergence (ecological speciation), or have rather been caused by post-speciation adaptation to diverse ecological opportunities, these results suggest that diverse ecological opportunities have likely facilitated the diversification of *Oxera* within New Caledonia. The decelerated diversification of pollination syndromes, dispersal niches, and life-forms (revealed by a rather high phylogenetic signal; Fig. 2c) likely indicates that all or most of these niches previously vacant were ultimately filled during the early history of the clade. Our comparative analyses indicate that from the late Pliocene to early Pleistocene, the major *Oxera* lineages (i.e., the seven New Caledonian subclades plus *O. morierei*) had already originated (Fig. 2a), most of them occupying a unique niche combination. It is important to note that all recovered *Oxera* subclades were robust to contrasting phylogenetic signal between nuclear and plastid genes (Supplementary Appendix S8 available on Dryad). Hence, each *Oxera* subclade robustly holds a unique trait combination (Table 4), excepting perhaps the *neriifolia* and *oblongifolia* subclades, which similarly exhibit slender lianas, bilabiate flowers and small rounded fruits. This evolutionary divergence in life forms seems not to be correlated with habitat divergence, since these two characteristics have evolved largely independently across *Oxera* (Fig. 2b and c), suggesting that different growth forms occupy distinct functional niches related to light requirements within habitats.

Our study thus detected strong ecological segregation among *Oxera* subclades and species (Fig. 1c), but the precision of our morphological data may not be sufficient to discriminate finer ecological syndromes encountered in *Oxera*. Compared with our results, the genus may exploit an even larger diversity in pollinators and dispersers than highlighted in this study. For example, the actinomorphic flowers of Pacific species are sometimes visited by honeyeaters (Belcher and Sibson 1982; de Kok 1997). Gullet flowers with small and narrow tubes, such as the ones of *O. sessilifolia*, *O. garoensis* sp. nov. ined., and *O. doubetiae* sp. nov. ined., may be more efficiently pollinated by Diptera or Coleoptera rather than birds (Jourdan, pers. comm.). It would be illuminating to conduct detailed investigations on the pollination biology of different *Oxera* species using more precise measurements of floral morphology characters. This could reveal that pollination syndromes have in fact spurred diversification during the evolution of *Oxera*.

### Recent Diversification Driven by the Interplay of Habitat Shifts and Allopatry

Our comparative analyses further suggest that since the end of Pliocene and the onset of the Pleistocene, the diversification of *Oxera* has been impacted by several punctual shifts in habitat preference (Fig. 2b and c). These events coincided with another intense period of soil morphogenesis and weathering events in New Caledonia which occurred between 3 and 2.5 Ma (Fig. 2a and b; Chevillotte et al. 2006), as well as several climatic oscillations documented in the south-western Pacific (Dodson and Macphail 2004). These events have likely caused myriad spatial rearrangements of New Caledonian biotas (Hope and Pask 1998, Stevenson et al. 2001, Stevenson 2004; Stevenson and Hope 2005), thus generating novel ecological opportunities, and hence the recent diversification of *Oxera*.

Phylogenetic patterns of geographic range overlap and asymmetry between pairs of species show that geographical isolation seems to have played a prominent role during the diversification of *Oxera*. This suggests that neutral divergence may have been at least as important as ecological speciation. Most species pairs within *Oxera* have clear allopatric distributions (null range overlap more frequent through all species pairs; Fig. 3). This is exemplified by the *baladica* subclade, which appears to have diversified almost entirely in allopatry (Fig. 4). Few species pairs clearly originated from sympatric speciation, as suggested by complete range overlaps and trait divergence (Figs. 3 and 4). Except for the *oblongifolia* subclade, the geographical range overlap between species remains remarkably low ($<$0.4; Supplementary Appendix S19 available on Dryad) indicating that given the recent radiations of *Oxera*, most sister species have remained in allopatry, and that secondary sympatry may not have yet occurred in most sister species pairs.

The examination of geographic and ecological overlap between species pairs suggests that local species coexistence may in fact be very rare in *Oxera*. Among the 96 putative pairs of species (within the seven New Caledonian subclades), only five pairs potentially coexist locally, although this has never been noted after years of field observations (Gâteblé, pers. obs.). For example in the *oblongifolia* subclade (which exhibits largely sympatric species), each species grows in a specific set of environmental conditions, excepting the ubiquist *O. oblongifolia* (Fig. 4). Contemporary species coexistence is probably prevented by the recent interplay of allopatry and parapatry due to habitat shifts and environmental oscillations that have caused cycles of forest expansion and retraction. With *Oxera* being mainly a forest-adapted group (25 of 38 species confined to forests, and forest habitat identified as ancestral; Supplementary Appendix S17 available on Dryad), it is likely that vegetation cycles along the complex topographical gradients of New Caledonia have triggered spatial divergence and probably speciation. This process has been further amplified by the limited dispersal ability of *Oxera* since its dispersers are relatively sedentary. Such processes of vegetation oscillations along steep physical gradients, sometimes creating barriers to dispersal, is now considered to be a major driver of clade diversification, as demonstrated in recent theoretical simulations (Aguilée et al. 2012).

## Conclusion: Is *OXERA* a Clear Adaptive Radiation from New Caledonia?

The genus *Oxera* constitutes a remarkable example of a rapid diversifying lineage that originated from a single ancestor. We observed that the group experienced an early burst of species diversification, followed by an exceptionally high and constant species diversification rate (Fig. 2b). This signature is considered typical of adaptive radiations for some authors (Guyer and Slowinski 1993; Slowinski and Guyer 1993; Soulebeau et al. 2015) but not all (Givnish 1997, 2015), mainly because it is usually thought that early diversification bursts are driven by the exploitation of new ecological opportunities, which allows lineages to enter new adaptive zones. The early history of *Oxera* seems to have been paved by niche shifts toward novel pollination, dispersal and life-form traits. These niche shifts later decelerated, perhaps due to a saturation of available niche space. However, our analyses do not highlight any diversity dependent diversification processes, which are often considered as another hallmark of adaptive radiations (Rabosky 2009). This is likely because other ecological opportunities have helped further diversification of the group after its initial phase of biotic niche exploration. More recent opportunities likely emerged through landscape formation and environmental oscillations which may have triggered allopatric and parapatric speciation processes. In fact, the explosive diversification of *Oxera* within New Caledonia appears to have been triggered by varied ecological opportunities during the course of evolution. Adaptive radiation should not be narrowly defined through quantitative tests or by quantifying diversification tempos, since some well-known adaptive radiations have little or no effect on speciation, or even a negative effect (Givnish 2015), and are therefore more identified by high rates of morphological and ecological diversification (Givnish 1997; Sanderson 1998); this remains to be investigated for the *Oxera* diversification.

The explosive radiation experienced by *Oxera* thus constitutes a remarkable model for studying the drivers involved in the evolution of the New Caledonian flora, especially for recent epochs. The genus is an unclassifiable, multifaceted radiation seemingly triggered by both adaptive and neutral processes (adaptive, geographical, climatic, etc.), and driven by several classes of biotic and abiotic factors. *Oxera* could be therefore regarded as a partial adaptive radiation since changing ecological opportunities have contributed to its radiation, with an early diversification of biotic traits (life-form, pollination, dispersal), but more recently driven by the joint action of habitat changes and allopatric speciation leading to scant local coexistence between species. Although different diversification drivers may have been overlapping in time, the early stage of *Oxera* diversification could be then viewed as a leapfrog radiation (sensu Chase and Palmer 1997), where diversification drivers have relayed to one another at different time periods, but geographic processes may have later been an equally important driver of speciation. Indeed, past climatic history of New Caledonia has also acted as a major evolutionary force, with two pivotal events, a dramatic aridity at the early Pliocene leading to the release of newly vacant niches, and the accentuation of glacial-interglacial cycles from the early Pleistocene, causing repeated biota rearrangements. From our study, it is legitimate to ask whether any other case of adaptive radiation can be clearly demonstrated in old islands system such as New Caledonia, since the distinctive hallmark of past adaptive divergence is progressively blurred by more recent environmental fluctuations creating new ecological opportunities.

## Supplementary Material

Data available from the Dryad Digital Repository: http://dx.doi.org/10.5061/dryad.mm1ft40 for DNA alignment matrices and geographical variables. The resulting trees are available from TreeBASE (ID 23353).

## References

[B1] Aguilée R., Claessen D., Lambert A. 2012 Adaptive radiation driven by the interplay of eco-evolutionary and landscape dynamics. Evolution. 67:1291–1306. 23617909 10.1111/evo.12008

[B2] Aguilée R., Lambert A., Claessen D. 2011 Ecological speciation in dynamic landscapes. J. Evol. Biol. 24:2663–2677. 21954829 10.1111/j.1420-9101.2011.02392.x

[B3] Anacker B.L., Strauss S.Y. 2014 The geography and ecology of plant speciation: range overlap and niche divergence in sister species. Proc. R. Soc. B Biol. Sci. 281:20132980. 10.1098/rspb.2013.2980 PMC3906944 24452025

[B4] Baker W.J., Couvreur T.L.P. 2012 Global biogeography and diversification of palms sheds light on the evolution of tropical lineages. I. Historical biogeography. J. Biogeogr. 40:286–298.

[B5] Barrabé L., Karnadi-Abdelkader G., Ounémoa J., de Kok R.P.J., Robert N., Gâteblé G. 2015 Recircumscription of *Oxera* (Lamiaceae: Ajugoideae) to include *Faradaya* based on molecular and anatomical data. Bot. J. Linn. Soc. 179:693–711.

[B6] Barrabé L., Maggia L., Pillon Y., Rigault F., Mouly A., Davis A.P., Buerki S. 2014 New Caledonian lineages of *Psychotria* (Rubiaceae) reveal different evolutionary histories and the largest documented plant radiation for the archipelago. Mol. Phylogenet. Evol. 71:15–35. 24211193 10.1016/j.ympev.2013.10.020

[B7] Barré N., Villard P., Manceau N., Monimeau L., Ménard C. 2006 Les oiseaux de l’archipel des Loyauté (Nouvelle-Calédonie): inventaire et éléments d’écologie et de biogéographie. Revue d’Écologie 61:175–194.

[B8] von Beichle U. 1987 Lebensraum, Bestand und Nahrungsaufnahme der Zahntaube, *Didunculus strigirostris*. J. Orn. 128:75–89.

[B9] Belcher W.J., Sibson R.B. 1982 Birds of Fiji in colour. Auckand: Collins Publishers.

[B10] Bonvallot J. 2012 L’orohydrographie. In: Bonvallot J., Gay J.-C., Habert E., editors. Atlas de la Nouvelle-Calédonie. Marseille-Nouméa: IRD-Congrès de la Nouvelle-Calédonie p. 25–28.

[B11] Carlquist S.J., Baldwin B.G., Carr G.D. 2003 Tarweeds & Silverswords: evolution of the Madiinae (Asteraceae). St Louis, MO: Missouri Botanical Garden Press.

[B12] Carpenter R.J., Read J., Jaffré T. 2003 Reproductive traits of tropical rain-forest trees in New Caledonia. J. Trop. Ecol. 19:351–365.

[B13] Chase M.W., Palmer J.D. 1997 Leapfrog radiation in floral and vegetative traits among twig epiphytes in the orchid subtribe Oncidiinae. In: Givnish T.J., Sytsma K.J., editors. Molecular evolution and adaptive radiation. Cambridge: Cambridge University Press p. 331–350.

[B14] Chessel D., Dufour A.B., Thioulouse J. 2004 The ade4 package—I: one-table methods. R News. 4:5–10.

[B15] Chevillotte V., Chardon D., Beauvais A., Maurizot P., Colin F. 2006 Long-term tropical morphogenesis of New Caledonia (Southwest Pacific): importance of positive epeirogeny and climate change. Geomorphology. 81:361–375.

[B16] Cibois A., Thibault J.-C., Pasquet E. 2017 Uniform phenotype conceals double colonization by reed-warblers of a remote Pacific archipelago. J. Biogeogr. 34:1150–1166.

[B17] Clark J.R., Ree R.H., Alfaro M.E., King M.G., Wagner W.L., Roalson E.H. 2008 A comparative study in ancestral range reconstruction methods: retracing the uncertain histories of insular lineages. Syst. Biol. 57:693–707. 18853357 10.1080/10635150802426473

[B18] Clark J.R., Wagner W.L., Roalson E.H. 2009 Patterns of diversification and ancestral range reconstruction in the southeast Asian-Pacific angiosperm lineage *Cyrtandra* (Gesneriaceae). Mol. Phylogenet. Evol. 53:982–994. 19751837 10.1016/j.ympev.2009.09.002

[B19] Cluzel D., Maurizot P., Collot J., Sevin B. 2012 An outline of the geology of New Caledonia; from Permian-Mesozoic Southeast Gondwanaland active margin to Cenozoic obduction and supergene evolution. Episodes. 35:72–86.

[B20] Colombo R. 2008 Stratégies de dispersion chez quelques espèces de Sapotaceae de Nouvelle-Calédonie. Implications pour la conservation des forêts humides. Nouméa: Institut de Recherche pour le Développement, Université de Montpellier II.

[B21] Cooper W., Cooper W.T. 2004 Fruits of the Australian tropical rainforest. Clifton Hill: Nokomis Publications.

[B22] Crisp M.D., Cook L.G., Steane D. 2004 Radiation of the Australian flora: what can comparisons of molecular phylogenies across multiple taxa tell us about the evolution of diversity in present-day communities? Phil. Trans. R. Soc. B. 359:1551–1571. 15519972 10.1098/rstb.2004.1528 PMC1693438

[B23] de Kok R.P.J. 1997 The biology and systematics of *Oxera*, *Faradaya* and *Hosea* (Labiatae) [D. Phil. Thesis]. [Oxford]: University of Oxford.

[B24] de Kok R.P.J. 1998 The systematics and pollination biology of Oxera Labill., Faradaya F. Muell. and Hosea Ridl. (Labiatae). In: Saw L.G., Chua L.S.L, Khoo K.C., editors. 4th International Flora Malesiana Symposium. Kuala Lumpur: Forest Research Institute Malaysia p. 87–91.

[B25] de Kok R.P.J., Mabberley D.J. 1999a Generic and intra-generic delimitation of *Oxera* Labill. (Labiatae). Kew Bulletin. 54:257–264.

[B26] de Kok R.P.J., Mabberley D.J. 1999b The genus *Faradaya F. Muell. (Labiatae)*. Blumea. 44:321–342.

[B27] Dodson J.R., Macphail M.K. 2004 Palynological evidence for aridity events and vegetation change during the Middle Pliocene, a warm period in Southwestern Australia. Glob. Planet. Change. 41:285–307.

[B28] Donoghue M.J., Edwards E.J. 2014 Biome shifts and niche evolution in plants. Annu. Rev. Ecol. Evol. Syst. 45:547–572.

[B29] Drummond A.J., Rambaut A. 2007 BEAST: Bayesian evolutionary analysis by sampling trees. BMC Evol. Biol. 7:214. 17996036 10.1186/1471-2148-7-214 PMC2247476

[B30] Fitzjohn R.G., Maddison W.P., Otto S.P. 2009 Estimating trait-dependent speciation and extinction rates from incompletely resolved phylogenies. Syst. Biol. 58:595–611. 20525612 10.1093/sysbio/syp067

[B31] Fitzpatrick B.M., Turelli M. 2006 The geography of mammalian speciation: mixed signals from phylogenies and range maps. Evolution. 60:601–615. 16637504

[B32] Foster C.S.P., Ho S.Y.W., Conn B.J., Henwood M.J. 2014 Molecular systematics and biogeography of *Logania* R.Br. (Loganiaceae). Mol. Phylogenet. Evol. 78:324–333 24929247 10.1016/j.ympev.2014.06.001

[B33] Gallagher S.J., Greenwood D.R., Taylor D., Smith A.J., Wallace M.W., Holdgate G.R. 2003 The Pliocene climatic and environmental evolution of southeastern Australia: evidence from the marine and terrestrial realm. Palaeogeogr. Palaeoclimatol. Palaeoecol. 193:349–382.

[B34] Gallaher T., Callmander M.W., Buerki S., Keeley S.C. 2015 A long distance dispersal hypothesis for the Pandanaceae and the origins of the *Pandanus tectorius* complex. Mol. Phylogenet. Evol. 83:20–32. 25463018 10.1016/j.ympev.2014.11.002

[B35] Gianoli E. 2004 Evolution of a climbing habit promotes diversification in flowering plants. Proc. Biol. Sci. 271:2011–2015. 15451690 10.1098/rspb.2004.2827 PMC1691831

[B36] Givnish T.J. 1995 Plant stems: biomechanical adaptation for energy capture and influence on species distributions. In: Gartner B.L., editor. Plant stems: physiology and functional morphology. San Diego, CA: Academic Press p. 3–49.

[B37] Givnish T.J. 1997 Adaptive radiation and molecular systematics: issues and approaches. In: Givnish T.J., Systma K.J., editors. Molecular evolution and adaptive radiation. Cambridge: Cambridge University Press p. 1–54.

[B38] Givnish T.J. 2010 Ecology of plant speciation. Taxon. 59:1326–1366.

[B39] Givnish T.J. 2015 Adaptive radiation versus ‘radiation’ and ‘explosive diversification’: why conceptual distinctions are fundamental to understanding evolution. New Phytol. 207:297–303. 26032979 10.1111/nph.13482

[B40] Givnish T.J., Millam K.C., Mast A.R., Paterson T.B., Theim T.J., Hipp A.L., Henss J.M., Smith J.F., Wood K.R., Sytsma K.J. 2009 Origin, adaptive radiation and diversification of the Hawaiian lobeliads (Asterales: Campanulaceae). Proc. Biol. Sci. 276:407–416. 18854299 10.1098/rspb.2008.1204 PMC2664350

[B41] Grossenbacher D.L., Veloz S.D., Sexton, J.P. 2014 Niche and range size patterns suggest that speciation begins in small, ecologically diverged populations in North American monkeyflowers (*Mimulus* spp.). Evolution. 68:1270–1280. 24433389 10.1111/evo.12355

[B42] Guyer C., Slowinski J.B. 1993 Adaptive radiation and the topology of large phylogenies. Evolution. 47:253–263. 28568105 10.1111/j.1558-5646.1993.tb01214.x

[B43] Harmon L.J., Weir J.T., Brock C.D., Glor R.E., Challenger W. 2008 GEIGER: investigating evolutionary radiations. Bioinformatics. 24:129–131. 18006550 10.1093/bioinformatics/btm538

[B44] Haxaire J., Salesne T. 2016 Description d’un nouveau Sphingidae du genre *Gnathothlibus* Wallengren, 1858 de Nouvelle-Calédonie (Lepidoptera, Sphingidae). The Eur. Entomol. 8:179–208.

[B45] He T., Lamont B.B., Fogliani B. 2016 Pre-Gondwanan-breakup origin of *Beauprea* (Proteaceae) explains its historical presence in New Caledonia and New Zealand. Sci. Adv. 2:e1501648. 27386508 10.1126/sciadv.1501648 PMC4928934

[B46] Heibl C., Calenge C. 2013 Phyloclim: integrating phylogenetics and climatic niche modelling. Available from: https://CRAN.R-project.org/package=phyloclim.

[B47] Hill M.O., Smith A.J.E. 1976 Principal component analysis of taxonomic data with multi-state discrete characters. Taxon. 25:249–255.

[B48] Hope G., Pask J. 1998 Tropical vegetational changes in the late Pleistocene of New Caledonia. Palaeogeogr. Palaeoclimatol. Palaeoecol. 142:1–21.

[B49] Huelsenbeck J.P., Nielsen R., Bollback J.P. 2003 Stochastic mapping of morphological characters. Syst. Biol. 52:131–158. 12746144 10.1080/10635150390192780

[B50] Hutson A.M., Mickelburgh S.P., Racey P.A. 1992 Old world fruit bats: an action plan for their conservation. Gland: IUCN.

[B51] Jaffré T. 1980 Etude écologique du peuplement végétal des sols dérivés de roches ultrabasiques en Nouvelle-Calédonie. Paris: ORSTOM.

[B52] Jaffré T. 1993 The relationship between ecological diversity and floristic diversity in New Caledonia. Biodiv. Lett. 1:82–87.

[B53] Johnson S.D. 2010 The pollination niche and its role in the diversification and maintenance of the Southern African flora. Phil. Trans. R. Soc. B. 365:499–516. 20047876 10.1098/rstb.2009.0243 PMC2838267

[B54] Jorgensen T.H., Olesen J.M. 2001 Adaptive radiation of island plants: evidence from *Aeonium* (Crassulaceae) of the Canary Islands. Perspect. Plant Ecol. Syst. 4:29–42.

[B55] Karas C., Nurnberg D., Tiedemann R., Garbe-Schonberg D. 2011 Pliocene climate change of the Southwest Pacific and the impact of ocean gateways. Earth Planet. Sci. Lett. 301:117–124.

[B56] Keppel G., Prentis P., Biffin E., Hodgskiss P., Tuisese S., Tuiwawa M.V., Lowe A.J. 2011 Diversification history and hybridisation of *Dacrydium* (Podocarpaceae) in remote Oceania. Aust. J. Bot. 59:262–273.

[B57] Kim S.-C., McGowen M.R., Lubinsky P., Barber J.C., Mort ME, Santos-Guerra A. 2008 Timing and tempo of early and successive adaptive radiations in Macaronesia. PLoS One. 3:e2139. 18478126 10.1371/journal.pone.0002139 PMC2367450

[B58] Knope M.L., Morden C.W., Funk V.A., Fukami, T. 2012 Area and the rapid radiation of Hawaiian *Bidens* (Asteraceae). J. Biogeogr. 39:1206–1216.

[B59] Kranitz M.L., Biffin E., Clark A., Hollingsworth M.L., Ruhsam M., Gardner M.F., Thomas P., Mill R.R., Ennos R.A., Gaudeul M., Lowe A.J., Hollingsworth P.M. 2014 Evolutionary diversification of new Caledonian *Araucaria*. PLoS One. 9:e110308. 25340350 10.1371/journal.pone.0110308 PMC4207703

[B60] Losos J.B. 2010 Adaptive radiation, ecological opportunity, and evolutionary determinism. Am. Nat. 175:623–639. 20412015 10.1086/652433

[B61] Losos J.B., Ricklefs R.E. 2009 Adaptation and diversification on islands. Nature. 457:830–836. 19212401 10.1038/nature07893

[B62] Magallón S., Sanderson M.J. 2001 Absolute diversification rates in Angiosperm clades. Evolution 55:1762–1780. 11681732 10.1111/j.0014-3820.2001.tb00826.x

[B63] Magallón S., Gómez-Acevedo S., Sánchez-Revees L.L., Hernández-Hernández T. 2015 A metacalibrated time-tree documents the early rise of flowering plant phylogenetic diversity. New Phytol. 207:437–453. 25615647 10.1111/nph.13264

[B64] Manns U., Wikström N., Taylor C.M., Bremer B. 2012 Historical biogeography of the predominantly Neotropical subfamily *Cinchonoideae* (Rubiaceae): into or out of America. Int. J. Plant Sci. 173:261–286.

[B65] Meehan H.J., McConkey K.R., Drake D.R. 2002 Potential disruptions to seed dispersal mutualisms in Tonga, Western Polynesia. J. Biogeogr. 29:695–712.

[B66] Momose K., Nagamitsu T., Inoue T. 1998 Thrips cross-pollination of *Popowia pisocarpa* (Annonaceae) in a lowland dipterocarp forest in Sarawak. Biotropica. 30:444–448.

[B67] Morat P. 1993 Our knowledge of the flora of New Caledonia: endemism and diversity in relation to vegetation types and substrates. Biodiv. Lett. 1:72–81.

[B68] Morat P., Jaffré T., Tronchet F., Munzinger J., Pillon Y., Veillon J.-M., Chalopin M. 2012 Le référentiel taxonomique Florical et les caractéristiques de la flore vasculaire indigène de la Nouvelle-Calédonie. Adansonia. 34:179–221.

[B69] Morlon H. 2014 Phylogenetic approaches for studying diversification. Ecol. Lett. 17:508–525. 24533923 10.1111/ele.12251

[B70] Munzinger J., Morat P., Jaffré T., Gâteblé G., Pillon Y., Tronchet F., Veillon J.-M., Chalopin M. 2016 FLORICAL: checklist of the vascular indigenous flora of New Caledonia. Version 22.IV.2016. Available from: http://www.botanique.nc/herbier/florical.

[B71] Nylander J.A.A., Ronquist F., Huelsenbeck J.P., Nieves-Aldrey J.L. 2004 Bayesian phylogenetic analysis of combined data. Syst. Biol. 53:47–67. 14965900 10.1080/10635150490264699

[B72] Ogutcen E. 2016 The effects of dispersal and pollination on Plantaginaceae diversification [D. Phil. Thesis]. [Calgary]: University of Calgary.

[B73] Pagel M. 1999 Inferring the historical patterns of biological evolution. Nature. 401:877–884. 10553904 10.1038/44766

[B74] Papadopulos A.S.T., Baker W.J., Crayn D., Butlin R., Kynast R.G., Hutton I., Savolainen V. 2011 Speciation with gene flow on Lord Howe Island. PNAS. 108:13188–13193. 21730151 10.1073/pnas.1106085108 PMC3156170

[B75] Paradis E., Claude J., Strimmer K. 2004 APE: analyses of phylogenetics and evolution in R language. Bioinformatics. 20:289–290. 14734327 10.1093/bioinformatics/btg412

[B76] Paun O., Turner B., Trucchi E., Munzinger J., Chase M.W., Samuel R. 2016 Processes driving the adaptive radiation of a tropical tree (*Diospyros*, Ebenaceae) in New Caledonia, a biodiversity hotspot. Syst. Biol. 65:212–277. 26430059 10.1093/sysbio/syv076 PMC4748748

[B77] Pauw A. 2013 Can pollination niches facilitate plant coexistence? Trends Ecol. Evol. 28:30–37. 22951227 10.1016/j.tree.2012.07.019

[B78] Pfeil B.E., Crisp M.D. 2008 The age and biogeography of *Citrus* and the orange subfamily (Rutaceae: Aurantioideae) in Australasia and New Caledonia. Am. J. Bot. 95:1621–1631. 21628168 10.3732/ajb.0800214

[B79] Pillon Y. 2012 Time and tempo of diversification in the flora of New Caledonia. Bot. J. Linn. Soc. 170:288–298.

[B80] Pillon Y., Barrabé L., Buerki S. 2017 How many genera of vascular plants are endemic to New Caledonia? A critical review based on phylogenetic evidence. Bot. J. Linn. Soc. 183:177–198.

[B81] Pillon Y., Hopkins M.S., Rigault F., Jaffré T., Stacy E. 2014 A. Cryptic adaptive radiation in tropical forest trees in New Caledonia. New Phytol. 202:521–530. 24443886 10.1111/nph.12677

[B82] Pillon Y., Munzinger J., Amir H., Lebrun M. 2010 Ultramafic soils and species sorting in the flora of New Caledonia. J. Ecol. 98:1108–1116.

[B83] Pintaud J.-C., Jaffré T., Puig H. 2001 Chorology of New Caledonian palms and possible evidence of Pleistocene rain forest refugia. C. R. Acad. Sci. III. 324:453–463. 11411288 10.1016/s0764-4469(01)01312-9

[B84] Posada D. 2008 jModelTest: phylogenetic model averaging. Mol. Biol. Evol. 25:1253–1256. 18397919 10.1093/molbev/msn083

[B85] Pouteau R., Trueba S., Feild T.S., Isnard S. 2015 New Caledonia: a Pleistocene refugium for rain forest lineages of relict angiosperms. J. Biogeogr. 42:2062–2077.

[B86] Quiroga M.P., Mathiasen P., Iglesias A., Mill R.R., Premoli A.C. 2016 Molecular and fossil evidence disentangle the biogeographical history of *Podocarpus*, a key genus in plant geography. J. Biogeogr. 43:372–383.

[B87] Rabosky D.L. 2009 Ecological limits and diversification rate: alternative paradigms to explain the variation in species richness among clades and regions. Ecol. Lett. 12:735–743 19558515 10.1111/j.1461-0248.2009.01333.x

[B88] Rabosky D.L., Grundler M., Anderson C., Title P., Shi J.J., Brown J.W., Huang H., Larson J.G. 2014 BAMMtools: an R package for the analysis of evolutionary dynamics on phylogenetic trees. Methods Ecol. Evol. 5:701–707.

[B89] Revell L.J. 2012 phytools: an R package for phylogenetic comparative biology (and other things). Methods Ecol. Evol. 3:217–223.

[B90] Ricklefs R.E., Renner S.S. 1994 Species richness within families of flowering plants. Evolution. 48:1619–1636 . 28568402 10.1111/j.1558-5646.1994.tb02200.x

[B91] Richards G.C. 1990 The spectacled flying-fox, *Pteropus conspicillatus* (Chiroptera: Pteropodidae), in north Queensland. 2. Diet, seed dispersal and feeding ecology. Aust. Mammal. 13:25–31.

[B92] Ronquist F., Teslenko M., van der Mark P., Ayres D.L., Darling A., Höhna S., Larget B., Liu L., Suchard M.A., Huelsenbeck J.P. 2012 MrBayes 3.2: efficient Bayesian phylogenetic inference and model choice across a large model space. Syst. Biol. 61:539–542. 22357727 10.1093/sysbio/sys029 PMC3329765

[B93] Rundell R.J., Price T.D. 2009 Adaptive radiation, nonadaptive radiation, ecological speciation and nonecological speciation. Trends Ecol. Evol. 24:394–399. 19409647 10.1016/j.tree.2009.02.007

[B94] Sanderson M.J. 1998 Reappraising adaptive radiation. Am. J. Bot. 85:1650–1655.

[B95] Santiago L.S., Wright S.J. 2007 Leaf functional traits of tropical forest plants in relation to growth form. Funct. Ecol. 21:19–27.

[B96] Sauquet H., Ho S.Y., Gandolfo M.A., Jordan G.J., Wilf P., Cantrill D.J., Baily M.J., Bromham G.K., Carpenter R.J., Lee D.M., Murphy D.J., Sniderman J.M., Udovicic F. 2012 Testing the impact of calibration on molecular divergence times using a fossil-rich group: the case of *Nothofagus* (Fagales). Syst. Biol. 61:289–313. 22201158 10.1093/sysbio/syr116

[B97] Sauquet H., Weston P.H., Anderson C.L., Barker N.P., Cantrill D.J., Mast A.R., Savolainen V. 2009 Contrasted patterns of hyperdiversification in Mediterranean hotspots. PNAS. 106:221–225. 19116275 10.1073/pnas.0805607106 PMC2629191

[B98] Schluter D. 2000 The ecology of adaptive radiation. Oxford: Oxford University Press.

[B99] Selaya N.G., Anten N.P.R. 2008 Differences in biomass allocation, light interception and mechanical stability between lianas and trees in early secondary tropical forest. Funct. Ecol. 22:30–39.

[B100] Silvertown J., Francisco-Ortega J., Carine M. 2005 The monophyly of island radiations: an evaluation of niche pre-emption and some alternative explanations. J. Ecol. 93:653–657.

[B101] Slowinski J.B., Guyer C. 1993 Testing whether certain traits have caused amplified diversification—an improved method based on a model random speciation and extinction. Am. Nat. 142:1019–1024. 19425946 10.1086/285586

[B102] Soulebeau A., Aubriot X., Gaudeul M., Rouhan G., Hennequin S., Haevermans T., Dubuisson J.-Y., Jabbour F. 2015 The hypothesis of adaptive radiation in evolutionary biology: hard facts about a hazy concept. Org. Divers. Evol. 15:747–761.

[B103] Stadler T. 2011 Inferring speciation and extinction processes from extant species data. PNAS. 108:16145–16146. 21930908 10.1073/pnas.1113242108 PMC3182734

[B104] Stevenson J. 2004 A late-Holocene record of human impact from the southwest coast of New Caledonia. Holocene. 14:888–898.

[B105] Stevenson J., Dodson J.R., Prosser I.P. 2001 A late Quaternary record of environmental change and human impact from New Caledonia. Palaeogeogr. Palaeoclimatol. Palaeoecol. 168:97–123.

[B106] Stevenson J., Hope G.A. 2005 Comparison of late Quaternary forest changes in New Caledonia and northeastern Australia. Quaternary Res. 64:372–383.

[B107] Swenson U., Nylinder S., Munzinger J. 2013 Towards a natural classification of Sapotaceae subfamily Chrysophylloideae in Oceania and Southeast Asia based on nuclear sequence data. Taxon. 62:746–770.

[B108] Tassin J., Boissenin M., Barré, N. 2010 Can *Ptilinopus greyii* (Columbidae) disperse seeds in New Caledonia’s dry forests? Pac. Sci. 64:527–532.

[B109] Theim T.J., Shirk R.Y., Givnish T.J. 2014 Spatial genetic structure in four understory *Psychotria* species (Rubiaceae) and implications for tropical forest diversity. Am. J. Bot. 101:1189–1199. 25002460 10.3732/ajb.1300460

[B110] Tobias J.A., Cornwallis C.K., Derryberry E.P., Claramunt S., Brumfield R.T., Seddon N. 2014 Species coexistence and the dynamics of phenotypic evolution in adaptive radiation. Nature. 506: 359–363. 24362572 10.1038/nature12874

[B111] Trail P.W. 1994 The phenology of rainforest plants in Tutuila, American Samoa. Biological Report Series 58 American Samoa Department of Marine & Wildlife Resources.

[B112] Turner B., Munzinger J., Duangjai S., Temsch E.M., Stockenhuber R., Barfuss M.H.J., Chase M.W., Samuel R. 2013 Molecular phylogenetics of New Caledonian *Diospyros* (Ebenaceae) using plastid and nuclear markers. Mol. Phylogenet. Evol. 69:740–763. 23850609 10.1016/j.ympev.2013.07.002 PMC3913082

[B113] Vitales D., Garnatje T., Pellicer J., Vallès J., Santos-Guerra A., Sanmartín I. 2014 The explosive radiation of *Cheirolophus* (Asteraceae, Cardueae) in Macaronesia. BMC Evol. Biol. 14:118. 24888240 10.1186/1471-2148-14-118 PMC4048045

[B114] Wagstaff S.J., Bayly M.J., Garnock-Jones, P.J., Albach D.C. 2002 Classification, origin, and diversification of the New Zealand hebes (Scrophulariaceae). Ann. Mo. Bot. Gard. 89:38–63.

[B115] Wagstaff S.J., Dawson M.I., Venter S., Munzinger J., Crayn D., Steane D.A., Lemson K.L. 2010 Origin, diversification, and classification of the Australasian genus *Dracophyllum* (Richeeae, Ericaceae). Ann. Mo. Bot. Gard. 97:235–258.

[B116] Wagstaff S.J., Garnock-Jones P.J. 1998 Evolution and biogeography of the *Hebe* complex (Scrophulariaceae) inferred from ITS sequences. N. Z. J. Bot. 36:425–437.

[B117] Warren D.L., Glor R.E., Turelli M. 2008 Environmental niche equivalency versus conservatism: quantitative approaches to niche evolution. Evolution. 62:2868–2883. 18752605 10.1111/j.1558-5646.2008.00482.x

[B118] Weeks A., Zapata F., Pell S.K., Daly D.C., Mitchell J.D., Fine P.V.A. 2014 To move or to evolve: contrasting patterns of intercontinental connectivity and climatic niche evolution in “Terebinthaceae” (Anacardiaceae and Burseraceae). Front. Genet. 5:409. 25506354 10.3389/fgene.2014.00409 PMC4247111

[B119] Whittaker R.J., Triantis K.A., Ladle R.J. 2008 A general dynamic theory of oceanic island biogeography. J. Biogeogr. 35:977–994.

[B120] Wotton D.M., Drake D.R., Powlesland R.G., Ladley J.J. 2016 The role of lizards as seed dispersers in New Zealand. J. R. Soc. N. Z. 46:40–65.

[B121] Wotton D.M., Kelly D. 2012 Do larger frugivores move seeds further? Body size, seed dispersal distance, and a case study of a large, sedentary pigeon. J. Biogeogr. 39:1973–1983.

[B122] Yang Y., Morden C.W., Sporck-Koehler M.J., Sack L., Wagner W.L., Berry P.E. 2018 Repeated range expansion and niche shift in a volcanic hotspot archipelago: Radiation of C4 Hawaiian Euphorbia subgenus *Chamaesyce* (Euphorbiaceae). Ecol. Evol. 8:8523–8536. 30250720 10.1002/ece3.4354 PMC6145001

[B123] Yao G., Drew B.T., Yi T.S., Yan H.F., Yuan Y.M., Ge X.J. 2016 Phylogenetic relationships, character evolution and biogeographic diversification of *Pogostemon* s.l. (Lamiaceae). Mol. Phylogenet. Evol. 98:184–200. 26923493 10.1016/j.ympev.2016.01.020

[B124] Yuan Y.-W., Mabberley D.J., Steane D.A., Olmstead R.G. 2010 Further disintegration and redefinition of *Clerodendrum* (Lamiaceae): implications for the understanding of the evolution of an intriguing breeding strategy. Taxon. 59:125–133.

[B125] Zachos J., Pagani M., Sloan L., Thomas E., Billups K. 2001 Trends, rhytms, and aberrations in global climate 65 Ma to present. Science. 292:686–693. 11326091 10.1126/science.1059412

